# Supplementary material for: Assessing the efficacy, safety and utility of 6-month day-and-night automated closed-loop insulin delivery under free-living conditions compared with insulin pump therapy in children and adolescents with type 1 diabetes: an open-label, multicentre, multinational, single-period, randomised, parallel group study protocol
Source: BMJ Open. 2019 Jun 3;9(6):e027856. doi: 10.1136/bmjopen-2018-027856 (PMC6561428; doi:10.1136/bmjopen-2018-027856)
Supplement: Author's manuscript [file bmjopen-2018-027856.draft_revisions.pdf]

BMJ Open is committed to open peer review. As part of this commitment we make the peer review history of every article we publish publicly available.

When an article is published we post the peer reviewers' comments and the authors' responses online. We also post the versions of the paper that were used during peer review. These are the versions that the peer review comments apply to.

The versions of the paper that follow are the versions that were submitted during the peer review process. They are not the versions of record or the final published versions. They should not be cited or distributed as the published version of this manuscript.

BMJ Open is an open access journal and the full, final, typeset and author-corrected version of record of the manuscript is available on our site with no access controls, subscription charges or pay-per-view fees (http://bmjopen.bmj.com).

If you have any questions on BMJ Open's open peer review process please email info.bmjopen@bmj.com

BMJ Open

Assessing the efficacy, safety and utility of 6 month day-and-night automated closed-loop insulin delivery under free living conditions compared to insulin pump therapy in children and adolescents with type 1 diabetes: a randomised parallel study protocol

Journal:	*BMJ Open*
Manuscript ID	bmjopen-2018-027856
Article Type:	Protocol
Date Submitted by the Author:	18-Nov-2018

Complete List of Authors:	Musolino, Gianluca; Wellcome Trust-MRC Institute of Metabolic Science, University of Cambridge; University of Cambridge, Department of Paediatrics<br>Allen, Janet; Wellcome Trust-MRC Institute of Metabolic Science, University of Cambridge ; University of Cambridge, Department of Paediatrics<br>Hartnell, Sara; Cambridge University Hospitals NHS Foundation Trust, Department of Diabetes and Endocrinology<br>Wilinska, Malgorzata; Wellcome Trust-MRC Institute of Metabolic Science, University of Cambridge ; University of Cambridge, Department of Paediatrics<br>Tauschmann, Martin; Wellcome Trust-MRC Institute of Metabolic Science, University of Cambridge; Medical University of Vienna, Department of Pediatrics and Adolescent Medicine<br>Boughton, Charlotte; Wellcome Trust-MRC Institute of Metabolic Science, University of Cambridge<br>Campbell, Fiona; Leeds Children's Hospital, Department of Paediatric Diabetes<br>Denvir, Louise; Nottingham University Hospitals NHS Trust, Department of Paediatric Diabetes and Endocrinology<br>Trevelyan, Nicola; Southampton Children's Hospital, Southampton General Hospital<br>Wadwa, Paul; Barbara Davis Center for Childhood Diabetes, University of Colorado<br>DiMeglio, Linda; Indiana University School of Medicine, Division of Pediatric Endocrinology and Diabetology and Wells Center for Pediatric Research, Department of Pediatrics<br>Buckingham, Bruce A.; Stanford University, Division of Pediatric Endocrinology<br>Weinzimer, Stuart; Yale University, Department of Pediatrics<br>Acerini, Carlo; Wellcome Trust-MRC Institute of Metabolic Science, University of Cambridge ; University of Cambridge, Department of Paediatrics<br>Hood, Korey; Stanford University, Division of Pediatric Endocrinology<br>Fox, Steven; University of Southern California, Department of Pharmaceutical and Health Economics, School of Pharmacy<br>Kollman, Craig; Jaeb Center for Health Research

Sibayan, Judy; Jaeb Center for Health Research
Borgman, Sarah; Jaeb Center for Health Research
Cheng, Peiyao; Jaeb Center for Health Research
Hovorka, Roman; Wellcome Trust-MRC Institute of Metabolic Science, University of Cambridge ; University of Cambridge, Department of Paediatrics

Keywords: Type 1 diabetes, Closed-loop, Artificial pancreas

SCHOLARONE™ Manuscripts

Assessing the efficacy, safety and utility of 6 month day-and-night automated closed-loop insulin delivery under free living conditions compared to insulin pump therapy in children and adolescents with type 1 diabetes: a randomised parallel study protocol.

[1,3]Musolino G, [1,3]Allen JM, [2]Hartnell S, [1,3]Wilinska ME, [1,4]Tauschmann M, [1]Boughton C, [5]Campbell F, [6]Denvir L, [7]Trevelyan N, [8]Wadwa RP, [9]DiMeglio LA, [10]Buckingham BA, [11]Weinzimer SA, [1,3]Acerini CL, [10]Hood KK, [12]Fox DS, [13]Kollman C, [13]Sibayan J, [13]Borgman S, [13]Cheng P, and [1,3]Hovorka R on behalf of DAN05 Consortium

[1]Wellcome Trust-MRC Institute of Metabolic Science, University of Cambridge, Cambridge, UK

[2]Department of Diabetes & Endocrinology, Cambridge University Hospitals NHS Foundation Trust, Cambridge, UK

[3]Department of Paediatrics, University of Cambridge, Cambridge, UK

[4]Department of Pediatrics and Adolescent Medicine, Medical University of Vienna, Austria

[5]Leeds Children's Hospital, Department of Paediatric Diabetes, Leeds, UK

[6]Department of Paediatric Diabetes and Endocrinology, Nottingham University Hospitals NHS Trust, Nottingham, UK

[7]Southampton Children's Hospital, Southampton General Hospital, Southampton, UK

[8]Barbara Davis Center for Childhood Diabetes, University of Colorado, Aurora, CO, USA

[9]Division of Pediatric Endocrinology and Diabetology and Wells Center for Pediatric Research, Department of Pediatrics, Indiana University School of Medicine, Indianapolis, IN, USA

[10]Division of Pediatric Endocrinology, Stanford University, Stanford, CA, USA

[11]Department of Pediatrics, Yale University, New Haven, CT, USA

[12]Department of Pharmaceutical and Health Economics, School of Pharmacy, University of Southern California, Los Angeles, CA, USA

[13]Jaeb Center for Health Research, Tampa, FL, USA

Correspondence

Roman Hovorka, PhD

University of Cambridge Metabolic Research Laboratories and NIHR Cambridge Biomedical Research Centre,

Wellcome Trust-MRC Institute of Metabolic Science, Box 289,

Addenbrooke's Hospital, Hills Road, Cambridge CB2 0QQ, UK

Tel: +44 1223 762 862, fax: +44 1223 330 598, e-mail: rh347@cam.ac.uk

Key Words: Type 1 diabetes, Closed-loop, Artificial pancreas

Word count abstract: 300

Word count paper: 4000

For peer review only - http://bmjopen.bmj.com/site/about/guidelines.xhtml

ABSTRACT

Introduction Closed-loop systems titrate insulin based on sensor glucose levels, providing novel means to reduce the risk of hypoglycaemia while improving glycaemic control. The present study will assess the effectiveness of 6-month day-and-night closed-loop insulin delivery compared to insulin pump therapy in children and adolescents with type 1 diabetes.

Methods and analysis The trial adopts an open-label, multi-centre, multi-national (UK and USA), randomised, single-period, parallel design. Participants (n=130) are children and adolescents (age ≥ 6 and <19 years) with type 1 diabetes for at least 1 year, and insulin pump use for at least 3 months with sub-optimal glycaemic control [glycated haemoglobin ≥58mmol/mol (7.5%) and ≤86mmol/mol (10%)]. After a 2-3 week run-in period, participants will be randomised (1:1 within each stratum) to 6-month use of hybrid closed-loop insulin delivery, or to insulin pump therapy. Analyses will be conducted on an intention to treat basis. The primary outcome is glycated haemoglobin at 6 months. Other key endpoints include time spent in the target glucose range (3.9 to 10mmol/l, 70 to 180mg/dl), mean sensor glucose, and time spent above and below target. Secondary outcomes include standard deviation and coefficient of variation of sensor glucose levels, time with sensor glucose levels <3.5mmol/l (63mg/dl) and <3.0mmol/l (54mg/dl), area under the curve of glucose <3.5mmol/l (63mg/dl), time with glucose levels >16.7mmol/l (300mg/dl), area under the curve of glucose >10.0mmol/l (180mg/dl), total, basal and bolus insulin dose, body mass index z-score, and blood pressure. Cognitive, emotional and behavioural characteristics of participants and family members and their responses to the closed-loop system and clinical trial will be assessed. An incremental cost-effectiveness ratio (ICER) for the closed-loop will be estimated.

Ethics and dissemination Ethics/institutional review board approval has been gained. The findings of this study will be disseminated by peer-review publications and conference presentations.

Trial registration NCT02925299 (ClinicalTrials.gov)

For peer review only - http://bmjopen.bmj.com/site/about/guidelines.xhtml

Strengths and limitations of this study

- The study adopts an open-label, multi-centre, multi-national, randomised, parallel design: it includes a large group of children and adolescents across wide geographical locations
- The trial adopts a 6-month follow-up period of hybrid closed-loop insulin delivery during unrestricted living
- Participants in the two study groups will have an equal number of study visits
- The study design excludes participants with recurrent incidents of severe hypoglycaemia or diabetic ketoacidosis during the previous 6 months, living alone, and those with glycated haemoglobin below 58mmol/mol (7.5%) and above 86mmol/mol (10%) and with high or very low daily insulin requirements (total daily insulin dose ≥2IU/kg/day or <15IU/day)
- All participants are already pump users, somewhat limiting generalizability

For peer review only - http://bmjopen.bmj.com/site/about/guidelines.xhtml

INTRODUCTION

Type 1 diabetes is characterised by a deficiency of insulin caused by immunologically-mediated damage to pancreatic beta cells, leading to raised blood glucose levels. Diabetes is one of the most common metabolic conditions in children and adults. It is estimated that in 2017 1,100,000 children and adolescents (0-19 years) worldwide had type 1 diabetes and that the number of newly diagnosed cases was over 130,000 (1). The incidence rate in children is increasing by approximately 3-4% per year with geographic differences (1). Earlier onset can result in diabetes complications appearing at a younger age, whilst dependence on lifelong insulin imposes a heavy burden on children, carers as well as health care systems.

Despite continuing progress, glycaemic control in children and adolescents with type 1 diabetes remains suboptimal (2). The achievement of recommended treatment goals is limited by the ever present risk of hypoglycaemia. Even in those with the desired level of glycaemic control, non-physiological glucose excursions occur with periods of silent hyper- and hypoglycaemia (3, 4). Individuals have blunted counter-regulatory responses to hypoglycaemia impairing recovery and increasing the threat of future episodes (5). Recurrent episodes may lead to hypoglycaemic unawareness, increasing the risk of severe hypoglycaemia (6). Hypoglycaemia has psychological consequences including the fear of hypoglycaemia with resulting maladaptive coping behaviours such as excessive eating or under-insulinising that may negatively impact glycaemic control (7).

The development of continuous glucose monitoring has been a major advance (8-11). Sensor-augmented pumps combine real-time continuous glucose monitoring with insulin pump (12). Insulin pumps with low glucose suspend feature have been shown to reduce hypoglycaemia (13). These systems, however, overall provide little or no automation to adjust insulin delivery to match glucose excursions.

An artificial pancreas (a closed-loop system) adjusts insulin automatically and represents a realistic treatment option for type 1 diabetes (14). The closed-loop control algorithm translates, in real-time, sensor glucose levels received from the glucose monitoring device and computes the amount of insulin to be delivered by the coupled insulin pump. Hybrid closed-loop systems automatically titrate insulin delivery although the user manages insulin boosts at meal time (15). In 2017, the first closed-loop system entered clinical use in the USA (16).

Closed-loop systems may improve glycaemic control while reducing the risk of hypoglycaemia (17). They have been evaluated in children and adolescents under controlled laboratory conditions (18-20) and in home settings (21-24). Investigations in adults have also been conducted (22, 25, 26). Psychosocial assessments support acceptability and benefits of this therapeutic approach among children/adolescents and carers (27). Closed-loop systems are associated with increased time in near normoglycaemia and reduced time in hypoglycaemia and hyperglycaemia (28). So far, evaluations have been limited to 3 months (22).

The present study will assess the efficacy, safety, utility and acceptability of 6-month day-and-night hybrid closed-loop insulin delivery during unrestricted living in comparison to insulin pump therapy in children and adolescents with type 1 diabetes.

For peer review only - http://bmjopen.bmj.com/site/about/guidelines.xhtml

METHODS AND ANALYSIS

Overview

This trial adopts an open-label, multi-centre, multi-national, single-period, randomised, parallel group design, involving a 6-month home study period during which day-and-night glucose levels will be managed either by a closed-loop system (intervention group) or by insulin pump therapy (control group) (Figure 1). We aim to recruit up to 150 children and adolescents aged ≥6 to <19 years with type 1 diabetes on insulin pump therapy (approximately equal proportion of those aged ≥6 to 12 years and 13 to <19 years, a minimum quota of 25% participants with baseline glycated haemoglobin >69mmol/mol, >8.5%). Inclusion and exclusion criteria are summarised in Table 1.

The University of Cambridge (UK) and Jaeb Center for Health Research (USA) are the coordinating centres. Clinical centres include:

1) Addenbrooke's Hospital, Cambridge, UK
2) Barbara Davis Center for Childhood Diabetes, Aurora, USA
3) Indiana University, Indianapolis, USA
4) Leeds Teaching Hospital, Leeds, UK
5) Nottingham Children's Hospital, Nottingham, UK
6) Southampton Children's Hospital, Southampton, UK
7) Stanford University, Stanford, California, USA
8) Yale University, New Haven, Connecticut, USA

Cognitive, emotional, and behavioural characteristics of participants and family members and their response to the closed-loop will be assessed gathering both quantitative (validated surveys) and qualitative data (focus groups). Written informed consent/assent will be obtained from all participants and guardians before any study-related activities.

Study schedule

The study will comprise up to 8 visits and 6 telephone/email contacts (see Table 2 and Table 3). The maximum study duration is 8 months.

Screening and baseline assessment

At screening, blood samples for full blood count, liver, thyroid function and anti-transglutaminase antibodies (with IgA levels if not done within previous 12 months) will be taken. Non-hypoglycaemia C-peptide, glucose and glycated haemoglobin will be measured and a urine pregnancy test in females of child-bearing potential will be performed. Surveys investigating participants' quality of life, psychosocial and cognitive functioning, and response to their current treatment will be distributed. Participants will be fitted with a blinded continuous glucose-monitoring device (Libre Pro, Abbott Diabetes Care, Alameda, CA, USA) that will be worn during the run-in period at home for up to 14 days.

Run-in period

During a 2-3 week run-in period, subjects will continue using their own insulin pump. Data obtained from blinded continuous glucose monitoring sensors and pump downloads may be utilised for treatment adjustments. The run-in period may be extended/repeated if no or

limited sensor data is available and/or if further optimisation is indicated. At least 10 days of sensor data need to be collected.

Randomisation

Central randomisation software will be used with stratification by site and baseline glycated haemoglobin. The randomisation ratio will be 1:1 within each stratum. The randomisation list created by the study statistician is encrypted.

Treatment period

1. Automated day and night hybrid closed-loop insulin delivery combined with low glucose suspend feature (interventional arm)

Participants allocated to the closed-loop group will be trained on using the study insulin pump (modified Medtronic 640G pump, investigational use only, Medtronic, Northridge, CA, USA) and real-time continuous glucose sensor (Guardian 3, Medtronic). Once deemed competent with the use of the devices, participants will receive training required for the closed-loop system. Competency on the use of closed-loop will be evaluated. During the closed-loop period, meal boluses will be programmed by the participant to be delivered by the insulin pump based on estimated ingested carbohydrate amounts. Specific instructions during closed-loop related to exercise management, sick day rules, hypo- and hyperglycaemia management and technical troubleshooting will be provided.

2. Usual care (conventional or sensor-augmented pump therapy) (control arm)

Participants in the control arm will receive refresher training on key aspects of insulin pump therapy. The use of capillary self-monitoring of blood glucose will be highlighted. During the 6-month control intervention period, subjects will continue using either their own insulin pump alone or combined with their pre-study glucose monitoring device.

At the end of the study initiation visit, participants in both study groups will be fitted with a blinded continuous glucose monitoring system (Libre Pro) that will be worn for up to 14 days. If the sensor fails or gets detached, another sensor may be inserted. The sensor data may be used to optimise insulin delivery.

Assessments at 3 months and 6 months

A blood sample will be collected for measurement of glycated haemoglobin. A urine pregnancy test in females of child-bearing potential will be performed. As per usual clinical practice glucometer downloads and pump data will be reviewed, and adjustments to insulin pump settings will be made as required. Validated surveys evaluating the impact of the devices employed on quality of life, psychosocial and cognitive functioning, diabetes management and treatment satisfaction will be administered. At the end of the 3-month follow-up visit, participants in both study groups will be fitted with blinded continuous glucose monitoring systems (Libre Pro). For assessment of glycaemic control during the final 3-month period of the trial, participants in both study groups will be fitted with a blinded continuous glucose monitoring system 2 to 4 weeks before the end of study. At the 6-month visit, the same procedures as at the 3-month visit will be followed. A subset of subjects/guardians will be invited to join follow-up focus groups.

For peer review only - http://bmjopen.bmj.com/site/about/guidelines.xhtml

Study contacts during 6-month study period

Participants in the two study groups will have an equal number of contact visits. The first planned contact will occur within 24-48 hours after study initiation visit. During the first 2 weeks of the study period, participants will be contacted weekly. Thereafter, participants will be contacted monthly. Subjects/parents and/or the clinical team are free to adjust insulin therapy as per usual clinical practice, but no active treatment optimisation will be undertaken by the research team.

Devices download

Participants will be invited to download insulin delivery and glucose data regularly from the insulin pump and blood glucose meter.

Closed-loop system

The FlorenceM closed-loop system (Figure 2) incorporates a computer-based algorithm hosted by an Android smartphone, which interacts wirelessly with the modified investigational-use-only 640G pump through a proprietary translator device included in the smartphone's enclosure. By using the information received from the glucose sensor every ten minutes, the system computes a new temporary basal insulin infusion rate, which is automatically sent to the insulin pump. The treat-to-target control algorithm aims to achieve a default glucose level of 5.8mmol/l (104mg/dl) and regulates the actual level depending on fasting versus postprandial status and the accuracy of model-based glucose predictions. No remote monitoring is planned. While the system is charging and connected to internet, the device uploads data on a server. The study pump comprises continuous glucose monitoring receiver and provides hypoglycaemia and hyperglycaemia alarms, which can be activated and personalised by the participants.

Safety precautions during closed-loop

Participants will be asked to perform capillary calibrations before breakfast and dinner. If sensor glucose value is >3.0mmol/l (54mg/dl) different from capillary glucose level, the sensor will be recalibrated. These directions are based on an in-silico simulation of hypo- and hyperglycaemia risk using the validated Cambridge simulator (29). If sensor glucose becomes unavailable, the pump will automatically deliver the pre-programmed insulin within 30 minutes. Safety rules limit maximum insulin infusion and suspend insulin delivery when sensor glucose is ≤4.3mmol/l (77mg/dl) or when sensor glucose is rapidly decreasing. In case of a communication failure between control algorithm device and the study pump, the low-glucose feature will interrupt insulin delivery, provided sensor glucose is available. Insulin delivery will be resumed in accordance of the low glucose suspend feature implemented on the study pump. A 24-hour local telephone helpline will be available for any technical device issues or problems related to diabetes management.

Participant withdrawal criteria

The following pre-randomisation withdrawal criteria will apply:

1. Subject/caregiver is unable to demonstrate safe use of study insulin pump as judged by the investigator

2. Subject/caregiver fails to demonstrate compliance with insulin pump and capillary self-monitoring of blood glucose during run-in

Pre- and post-randomisation withdrawal criteria will comprise:

3. Subjects/caregivers may terminate participation in the study at any time without necessarily giving a reason and without any personal disadvantage
4. Significant protocol violation or non-compliance
5. Two distinct episodes of severe hypoglycaemia
6. Two distinct episodes of diabetic ketoacidosis unrelated to infusion site failure and related to the use of the closed-loop
7. Decision by the investigator or the sponsor that termination is in the subject's best medical interest
8. Allergic reaction to insulin
9. Allergic reaction to adhesive surface of infusion set or glucose sensor
10. Subject becomes pregnant during the study period

Subjects withdrawn due to reasons 4-10 will be invited to provide blood sample at the end of the planned study intervention for the assessment of glycated haemoglobin.

Psychosocial evaluations

Cognitive, emotional, and behavioural characteristics of participating subjects and family members and their response to the closed-loop system and clinical trial will be assessed using validated surveys and focus groups. Surveys will be completed at baseline (prior to randomisation), at 3 and 6 months.

To assess how strongly participants value the benefits of the closed-loop (compared with the default insulin pump), we will conduct a discrete choice experiment (DCE). In the DCE, respondents will be asked to answer a series of binary choice questions (e.g., "Given a choice between option A or B, which would you prefer...") where those two options offer differing strengths and weaknesses. By varying the performance levels of these different desirable characteristics, we can assess their relative importance.

Focus groups will be completed at the end of the study (6 months). We will conduct virtual focus groups using HIPAA-approved software supported by Stanford University. Focus groups will be run with 3-6 participants and we will work from a script of open-ended questions used to gather feedback and reactions to the closed-loop system/insulin pump therapy, the clinical trial and quality of life changes. The participation of a moderator with advanced training will ensure consistency across groups. Sessions will be audio- and video-taped and transcribed by a professional transcription service.

Blood samples

Screening blood samples will be measured locally. Additional blood samples will be taken for the measurement of non-hypoglycaemia C-peptide and glycated haemoglobin at a central laboratory. Glycated haemoglobin will be assessed at baseline, 3, and 6 months. At each time point, glycated haemoglobin will be measured locally (clinical care) and centrally (analysis of study endpoints). The central analysis will be performed using an International Federation of Clinical Chemistry and Laboratory Medicine aligned method.

For peer review only - http://bmjopen.bmj.com/site/about/guidelines.xhtml

Patient and Public Involvement

The research question and study endpoints are based on feedback from participants of previous studies and in line with prioritising by stakeholders (30). The study design and the assessment of the burden of the intervention were reviewed by focus groups. Results will be disseminated to participants and general public through social media and will be made available on the sponsor's website.

Statistical analysis

Primary Outcome Analysis

The primary analysis will follow the intention-to-treat principle. Data from all randomised subjects will be analysed in the group to which the subjects were assigned through randomisation regardless of the actual treatment received. Data will not be truncated due to protocol deviations.

The primary analysis will evaluate between group differences in glycated haemoglobin levels at the end of treatment period. A 5% significance level will be considered statistically significant for the primary outcome comparison.

Means ± standard deviation (SD) values or percentiles appropriate to the distribution will be reported for the primary outcome by treatment group. The two treatment groups will be compared using a linear regression model adjusting for glycated haemoglobin at baseline, age, and clinical centre as random effect. A 95% confidence interval will be reported for the difference between the randomisation groups based on the linear regression model. Residual values will be examined for an approximate normal distribution. If values are highly skewed, then a transformation or robust statistical methods (e.g., non-parametric or MM estimation) will be used instead. A detailed analysis plan will be provided separately.

Other Key Endpoints

For the following key endpoints at 6 months, the familywise type I error rate will be controlled at two-sided $\alpha = 0.05$. A gatekeeping strategy will be used, where the primary endpoint will be tested first, if passing the significance testing, other key endpoints will be tested in the order listed below using the fixed-sequence method at $\alpha = 0.05$.

- Time spent in the target glucose range from 3.9 to 10.0mmol/l (70 to 180mg/dl)
- Mean sensor glucose
- Time spent above target glucose 10.0mmol/l (180mg/dl)
- Time spent below target glucose 3.9mmol/l (70mg/dl)

If a non-significant ($p > 0.05$) result is obtained for any outcome on this list, no further hypothesis testing will be performed for any metrics further down on the list.

Secondary Efficacy Analyses

For these exploratory analyses, the false discovery rate will be used to account for multiple comparisons:

<u>Continuous glucose monitoring derived indices</u>

- Standard deviation of sensor glucose
- Sensor glucose variability measured with the coefficient of variation

- The time with glucose <3.5mmol/l (63mg/dl)
- The time with glucose <3.0mmol/l (54mg/dl)
- Area under the curve of glucose below 3.5mmol/l (63mg/dl)
- The time spent in significant hyperglycaemia (glucose >16.7mmol/l, 300mg/dl)
- Area under the curve of glucose above 10.0mmol/l (180mg/dl)

The following sensor glucose metrics will also be calculated separately for day-time period (06:00-23:59) and night-time period (00:00-05:59):

- The time with sensor glucose from 3.9 to 10.0mmol/l (70-180mg/dl)
- Mean glucose
- Glucose variability as measured by standard deviation
- The time with sensor glucose <3.5mmol/l (63mg/dl)

<u>Binary metrics for glycated haemoglobin</u>

- HbA1c <53mmol/mol (7.0%)
- HbA1c <58mmol/mol (7.5%)
- Relative reduction ≥10% from baseline
- Absolute reduction ≥0.5% from baseline
- Absolute reduction ≥1% from baseline
- Absolute reduction ≥1% from baseline or HbA1c <53mmol/mol (7.0%)

<u>Insulin and other endpoints</u>

- Total, basal and bolus insulin dose
- Body weight (BMI z-score)
- Blood pressure

The above described glycaemic metrics will be based on sensor glucose levels collected during post-randomisation periods of blinded sensors wear.

Safety analyses

The following events will be recorded and compared between treatment groups:

- Number of severe hypoglycaemia events per subject and incidence rate per 100 person-years
- Number of diabetic ketoacidosis events per subject and incidence rate per 100 person-years
- Sensor glucose-measured hypoglycaemic events per week (>15 minutes with glucose <3mmol/l, 54mg/dl)
- Sensor glucose-measured hyperglycaemic events per week (>15 minutes with glucose >16.7mmol/l, 300mg/dl)
- Proportion of subjects with worsening of glycated haemoglobin from baseline to 6 months by >0.5%

If we record enough observed events to allow formal statistical modelling for above safety outcomes, we will perform the following analyses. Poisson regression models will be constructed to compare the treatment group difference for event rates by adjusting for age, baseline glycated haemoglobin and random site effect. If any outlier exists, a robust Poisson

regression model will be used instead. For binary glycated haemoglobin outcome, logistic regression models will be used to compare the treatment group difference by adjusting for age, baseline glycated haemoglobin and random site effect.

Utility assessments

The following system use/function outcomes in the intervention arm will be tabulated:

- Number of low glucose suspend events
- Percentage of time when closed-loop system use is functioning
- Percentage of time when continuous glucose monitoring is used

Subgroup analyses

No subgroups were considered during the power calculations. Interpretation of any subgroup analyses will depend on whether the overall analysis demonstrates a significant treatment group difference. In the absence of such difference, if performed, the subgroup analyses will be interpreted with caution.

Psychosocial analyses

Quantitative data on usability and satisfaction will be analysed using simple descriptive statistics. Additionally, we will analyse scores from the cognitive, emotional, and behavioural assessments to determine if changes occur over time and between groups.

We will construct predictive models in the general linear framework to examine the associations with primary outcomes. For the discrete choice experiment (DCE), the strength of preference (importance) of each performance attribute will be estimated from the pooled DCE responses using standard regression analysis techniques.

Qualitative data will be analysed using Atlas.ti (release 6.0; Scientific Software Development GmbH, Berlin, Germany) to organize and manage the entire corpus of focus group data.

Cost utility analyses

To inform reimbursement and other policy decision-making, we will conduct a cost utility analysis on the benefits of closed-loop. The analysis timeframe for both costs and benefits will include not just the study period, but also anticipated future impacts. Costs will be denominated in US Dollars. They will be framed to include both health-related expenditures and any realised or projected incremental health cost savings. Utility will be quantified in quality adjusted life years (QALYS). We will elicit health related quality of life (HRQOL) during the study period using two preference based measures of health status: the Child Health Utility 9D (31) and the EuroQol 5D-Y (32). Future health and cost impacts, beyond the study period, will be estimated using numerical modelling. Incremental cost effectiveness ratios, comparing the closed-loop system to usual care will be calculated.

Interim analysis

We will not perform an interim analysis.

For peer review only - http://bmjopen.bmj.com/site/about/guidelines.xhtml

Per-protocol analysis

We will conduct a per-protocol analysis in order to replicate the primary analysis, but limited to participants who did not withdraw from the study (withdrawals excluded even if they return for a 6-month glycated haemoglobin measurement) and used closed-loop for at least 70% of the time (intervention group).

Power calculation

Data from the JDRF Continuous Glucose Monitoring Randomised Clinical Trial (33) from subjects who would have met the eligibility criteria for the current trial were used to project the distribution of baseline and 6-month glycated haemoglobin. Among N=53 subjects meeting the eligibility criteria in the JDRF CGM RCT (n=20 subjects 8 to 12 years of age and n=33 subjects 13 to 18 years of age), the upper limit of the confidence interval for the effective SD of glycated haemoglobin was 0.71%. With this effective SD, for a true 0.4% reduction in glycated haemoglobin, power = 85%, 2-sided type 1 error = 5%, 1:1 randomisation, total sample size is estimated to be 116. Adding 10% for potential dropout/non-compliance results in a final total sample size of approximately 128 (64 in each treatment group).

STUDY MANAGEMENT

Data Safety Monitoring Board

A Data Safety Monitoring Board (DSMB) will be instituted. The DSMB will be notified of all serious adverse events and any unanticipated adverse device effects/events and will perform regular safety data review. The DSMB will report to the National Institute of Diabetes and Digestive and Kidney Diseases (the Funder) any safety concerns and recommendations for suspension or early termination of the trial.

Study sponsors

In the UK the study sponsors are the University of Cambridge and the Cambridge University Hospitals NHS Foundation Trust. Study sponsor in the USA is the Jaeb Center for Health Research.

Study management committee

A study management committee composed of the Chief Investigator, Study Coordinators, and Study Data Manager will meet monthly to discuss the operational aspects of the trial.

Data management and monitoring

Designated personnel from the Coordinating Centres will be responsible for maintaining quality assurance and quality control systems to ensure that the clinical portion of the trial is conducted and data are generated, documented and reported in compliance with the protocol, Good Clinical Practice, and the regulatory requirements.

We will observe confidentiality of subject data. Personal details for each participant with a link to a unique identification number will be held locally on a study screening log in the Trial Master File at each of the investigation centres. These details will not be disclosed at any other stage during the study, and all individual results will remain anonymous.

For peer review only - http://bmjopen.bmj.com/site/about/guidelines.xhtml

Indemnity

Indemnity for any harm rising on the conduct of research will be provided according to arrangements in respective countries:

1) UK - any liability arising from study design will be covered by the clinical trial insurance policy organised by the University of Cambridge. National Health Service indemnity cover will apply for any claims arising from management and conduct of research.

2) USA - any liability arising from study design will be under the responsibility of the participants or their insurance company.

ETHICS AND DISSEMINATION

Approval from independent Research Ethics Committee/Institutional Review Board has been obtained in the UK and the USA. The study has undergone a review by regulatory authorities in the UK (Medicines and Healthcare products Regulatory Agency) and in the USA (Food and Drug Administration). All participants will be provided with oral and written information about the trial and procedures involved in the study before obtaining written informed consent. For minors, parents/guardians will provide written informed consent, and written assent will be gained.

Standard operating procedures for monitoring and reporting of all adverse events and adverse device effects will be in place including serious adverse events, serious adverse device effects and specific adverse events, such as severe hypoglycaemia and significant hyperglycaemia with ketosis.

Any substantial amendments to the protocol and other documents shall be submitted to, and approved by, the independent Research Ethics Committee and Institutional Review Board (UK, East of England-Cambridge South Research Ethics Committee, #16/EE/0380; USA, Jaeb Center for Health Research Institutional Review Board certified by the Office for Human Research Protections, FWA #00000024) and the regulatory authorities, prior to implementation as per nationally agreed guidelines.

The study started enrolling participants in June 2017. The study is expected to complete clinical follow up by November 2019 and to report results in 2020. The trial results will be disseminated in internationally peer-reviewed scientific journals.

For peer review only - http://bmjopen.bmj.com/site/about/guidelines.xhtml

Acknowledgements

Jasdip Mangat supported development and validation of closed-loop system. Josephine Hayes, Matthew Haydock and Nicole Ashcroft (Institute of Metabolic Science, University of Cambridge) provided administrative support. NIHR Cambridge Clinical Research Facility will support the research team in their research-related activities. Artificial Pancreas focus group contributors provided feedback on the study design. West Midlands Young Persons Advisory Group reviewed Participant Information Sheets.

Contributors

RH, FC, LD, NT, RPW, LADM, BAB, SAW, CLA, and KKH co-designed the study. RH designed and implemented the glucose controller. GM and RH wrote the manuscript. All authors critically reviewed the report. No writing assistance was provided.

Funding

National Institute of Diabetes and Digestive and Kidney Diseases of the National Institutes of Health under Award Number UC4DK108520. Additional support for the artificial pancreas work is from National Institute for Health Research Cambridge Biomedical Research Centre, and Wellcome Strategic Award (100574/Z/12/Z). Medtronic is supplying discounted CGM devices, sensors, and details of communication protocol to facilitate real-time connectivity. Abbott Diabetes Care is providing Libre Pro sensors.

Relevant disclosures

RH reports having received speaker honoraria from Eli Lilly and Novo Nordisk, serving on advisory panel for Eli Lilly and Novo Nordisk, receiving licence fees from BBraun and Medtronic. RH and MEW report patent patents and patent applications.

MT has received speaker honoraria from Medtronic and NovoNordisk.

RPW reports receiving speaker honoraria from Dexcom and serving on advisory panels for Eli Lilly and Novo Nordisk and research support from Bigfoot Biomedical, Dexcom, Lexicon, Mannkind and Novo Nordisk. BAB is on Advisory Boards for Novo-Nordisk and Convatec, has received research support from Medtronic Diabetes, Tandem Diabetes, Insulet, Convatec, and Dexcom. SAW has received speaker honoraria from Medtronic, Insulet, and Tandem, and has received consultant honoraria from Sanofi and Zealand Pharmaceuticals.

KKH has received research support from Dexcom, Inc for an investigator-initiated project; he has received consultant fees from Lilly Innovation Center, Bigfoot Biomedical, and Insulet, Inc. LADM reports grants from Medtronic.

GM, JMA, SH, CB, FC, LD, NT, CLA, SF, CK, JS, SB, PC declare no competing financial interests exist.

Ethics and IRB approvals

East of England - Cambridge South Research Ethics Committee (UK), Jaeb Center for Health Research Institutional Review (IRB) Office (813-975-8690 or irb@jaeb.org) (USA).

Provenance and peer review

Not commissioned, internally peer reviewed.

Table 1. Inclusion and exclusion criteria

**Summary of inclusion criteria**
▪ Age ≥6 and <19 years
▪ Type 1 diabetes as defined by World Health Organization (34) for at least 1 year
▪ Use of an insulin pump for at least 3 months, with good knowledge of insulin self-adjustment by subject or caregiver as judged by the investigator
▪ Using U-100 rapid acting insulin analogues Aspart or Lispro only
▪ Willing to perform regular finger-prick blood glucose monitoring, with at least 4 blood glucose measurements per day
▪ Screening glycated haemoglobin ≥58 mmol/mol (7.5%) and ≤86mmol/mol (10%) based on analysis from local laboratory
▪ Literate in English
▪ Willing to wear continuous glucose sensor and closed-loop system at home
▪ Willing to follow study specific instructions
▪ Willing to upload pump and glucose sensor data at regular intervals
▪ Access to Wi-Fi
▪ Living with someone who is trained to administer glucagon and is able to seek emergency assistance
**Summary of exclusion criteria**
▪ Living alone
▪ Current use of any closed-loop system
▪ Any other physical or psychological disease likely to interfere with the normal conduct of the study and interpretation of the study results as judged by the investigator
▪ Untreated coeliac disease, adrenal insufficiency, or untreated thyroid disease
▪ Current treatment with drugs known to interfere with glucose metabolism, e.g., systemic corticosteroids, non-selective beta-blockers and monoamine oxidase inhibitors, etc.
▪ Known or suspected allergy to insulin
▪ Clinically significant nephropathy (estimated glomerular filtration rate <45ml/min) or on dialysis, neuropathy or active retinopathy (presence of maculopathy or proliferative changes) as judged by the investigator
▪ Recurrent incidents of severe hypoglycaemia (>1 episode) during the previous 6 months (adolescents: severe hypoglycaemia is defined as an event requiring assistance of another person to actively administer carbohydrates, glucagon, or take other corrective actions including episodes of hypoglycaemia severe enough to cause unconsciousness, seizures or attendance at hospital; children: severe hypoglycaemia is defined as an event associated with a seizure or loss of consciousness)
▪ Recurrent incidents of diabetic ketoacidosis (>1 episode) during the previous 6 months
▪ Unwilling to avoid regular use of acetaminophen
▪ Lack of reliable telephone facility for contact
▪ Total daily insulin dose ≥2 IU/kg/day and <15 IU/day
▪ Pregnancy, planned pregnancy, or breast feeding
▪ Severe visual or hearing impairment
▪ Seizure disorder
▪ Medically documented allergy towards the adhesive (glue) of plasters or unable to tolerate tape adhesive in the area of sensor placement
▪ Serious skin diseases (e.g., psoriasis vulgaris, bacterial skin diseases) located at places of the body likely to be used for localisation of the glucose sensor
▪ Abusing illicit drugs, prescription drugs or alcohol
▪ Use of pramlintide (Symlin), or other non-insulin glucose lowering agents including sulphonylureas, biguanides, DPP4-Inhibitors, GLP-1 analogues, SGLT-1/ 2 inhibitors at time of screening
▪ Shift work with working hours between 10pm and 8am
▪ Sickle cell disease, haemoglobinopathy, or has received red blood cell transfusion or erythropoietin within 3 months prior to time of screening
▪ Eating disorder such as anorexia or bulimia
▪ Employed by Medtronic Diabetes or with immediate family members employed by Medtronic Diabetes

Table 2. Schedule of study visits / phone contacts when the participant is randomised to day-and-night closed-loop combined with low glucose feature (intervention group)

	Visit/ contact	Description	Start relative to previous / next Visit / Activity	Duration
**Run-in**	**Visit 1**	Recruitment visit: consent, HbA1c, screening bloods, urine pregnancy test, baseline surveys, blinded CGM training and insertion		1-4 hours
	**Visit 2**	Review of baseline bloods, pump settings and CGM data; adjustment of treatment	2 weeks after Visit 1 (+1 week); Run-in could be repeated	1-2 hours
**Training Period**	**Visit 3**	Randomisation, repeat HbA1c if Visit 3 and Visit 1 are >28 days apart, urine pregnancy test, study pump training and initiation, competency assessment	May coincide with Visit 2, within 8 weeks of Visit 1	3-4 hours
	**Visit 3a**	Real-time CGM training and initiation, competency assessment	Within 0 to 7 days of Visit 3 (Visit 3a may coincide with Visit 3; training visits can be repeated)	2-4 hours
**CL + LGS Intervention (6 months)**	**Visit 4***	CL initiation at clinic/home: data download, CL and low glucose feature training, competency assessment, blinded CGM	4 weeks after Randomisation (±1 week)	2-6 hours
	Contact 1	Review use of study devices; study update	Within 24 to 48 hours after Visit 4	<1 hour
	**Visit 5****	Review use of study devices; study update	1 week after Visit 4 (± 3 days)	<1 hour
	Contact 2	Review use of study devices; study update	2 weeks after Visit 4 (±3 days)	<1 hour
	Contact 3	Review use of study devices; study update	1 month after Visit 4 (±2 weeks)	<1 hour
	Contact 4	Review use of study devices; study update	2 months after Visit 4 (±2 weeks)	<1 hour
	**Visit 6**	3-month visit: HbA1c, urine pregnancy test, data download, blinded CGM, surveys	4 months after Randomisation (±2 weeks)	1-3 hours
	Contact 5	Review use of study devices; study update	5 months after Randomisation (±2 weeks)	<1 hour
	Contact 6	Review use of study devices; study update	6 months after Randomisation (±2 weeks)	<1 hour
	**Visit 7**	Blinded CGM	2-4 weeks before planned Visit 8	<0.5 hour
	**Visit 8**	End of closed-loop treatment arm (6 months of CL): HbA1c, data download, surveys and focus groups; resume usual pump therapy	7 months after Randomisation (±2 weeks)	1-3 hours

* In-person clinic visit mandatory in USA only.
** Could be done via phone/e-mail in UK. In-person visit mandatory in USA only.
HbA1c, glycated haemoglobin; CGM, continuous glucose monitoring; CL, closed-loop.

For peer review only - http://bmjopen.bmj.com/site/about/guidelines.xhtml

Table 3. Schedule of study visits / phone contacts when the participant is randomised to usual care (conventional or sensor-augmented insulin pump therapy) (control group)

	Visit/ contact	Description	Start relative to previous / next Visit / Activity	Duration
**Run-in**	**Visit 1**	Recruitment visit: consent, HbA1c, screening bloods, urine pregnancy test, baseline surveys, blinded CGM training and insertion		1-4 hours
	**Visit 2**	Review of baseline bloods, pump settings and CGM data; adjustment of treatment	2 weeks after Visit 1 (+1 week); Run-in could be repeated	1-2 hours
**Training period**	**Visit 3**	Randomisation, repeat HbA1c if Visit 3 and Visit 1 are >28 days apart, urine pregnancy test, insulin pump refresher training, competency assessment	May coincide with Visit 2, within 8 weeks of Visit 1	3-4 hours
**Usual insulin pump therapy Intervention (6 months)**	**Visit 4***	Initiation of standard therapy arm at clinic/home, glucometer download, recording of current insulin requirements, blinded CGM	4 weeks after Randomisation (±1 week)	2-6 hours
	Contact 1	Study update	Within 24 to 48 hours after Visit 4	<1 hour
	**Visit 5****	Study update	1 week after Visit 4 (±3 days)	<1 hour
	Contact 2	Study update	2 weeks after Visit 4 (±3 days)	<1 hour
	Contact 3	Study update	1 month after Visit 4 (±2 weeks)	<1 hour
	Contact 4	Study update	2 months after Visit 4 (±2 weeks)	<1 hour
	**Visit 6**	3-month visit: HbA1c, urine pregnancy test, glucometer download, recording of current insulin requirements, surveys, blinded CGM	4 months after Randomisation (±2 weeks)	1-3 hours
	Contact 5	Study update	5 months after Randomisation (±2 weeks)	<1 hour
	Contact 6	Study update	6 months after Randomisation (±2 weeks)	<1 hour
	**Visit 7**	Blinded CGM	2-4 weeks before planned Visit 8	<0.5 hour
	**Visit 8**	End of standard pump therapy treatment arm (6 months): HbA1c, glucometer download, recording of current insulin requirements, surveys and focus groups, resume usual care	7 months after Randomisation (±2 weeks)	1-3 hours

* In-person clinic visit mandatory in USA only.
** Could be done via phone/e-mail.
HbA1c, glycated haemoglobin; CGM, continuous glucose monitoring.

For peer review only - http://bmjopen.bmj.com/site/about/guidelines.xhtml

Figure Legends

Figure 1 Study flow chart. HbA1c, glycated haemoglobin; CGM, continuous glucose monitoring.

Figure 2 FlorenceM closed-loop system prototype.

The system consists of a continuous glucose monitoring transmitter with Guardian 3 sensor (Medtronic), an insulin pump (modified 640G pump, Medtronic) and an Android smartphone running the control algorithm (Cambridge).

References

1. International Diabetes Federation. IDF Diabetes Atlas 2017. 8th Edition 2017 http://www.diabetesatlas.org (last accessed 26th July 2018).
2. Wood JR, Miller KM, Maahs DM, Beck RW, DiMeglio LA, Libman IM, et al. Most youth with type 1 diabetes in the T1D Exchange Clinic Registry do not meet American Diabetes Association or International Society for Pediatric and Adolescent Diabetes clinical guidelines. Diabetes Care. 2013;36(7):2035-7.
3. Diabetes Research in Children Network Study G, Buckingham B, Beck RW, Tamborlane WV, Xing D, Kollman C, et al. Continuous glucose monitoring in children with type 1 diabetes. J Pediatr. 2007;151(4):388-93, 93 e1-2.
4. Bode BW, Schwartz S, Stubbs HA, Block JE. Glycemic characteristics in continuously monitored patients with type 1 and type 2 diabetes: normative values. Diabetes Care. 2005;28(10):2361-6.
5. Cryer PE. Diverse causes of hypoglycemia-associated autonomic failure in diabetes. N Engl J Med. 2004;350(22):2272-9.
6. Cryer PE. Mechanisms of hypoglycemia-associated autonomic failure and its component syndromes in diabetes. Diabetes. 2005;54(12):3592-601.
7. Wild D, von Maltzahn R, Brohan E, Christensen T, Clauson P, Gonder-Frederick L. A critical review of the literature on fear of hypoglycemia in diabetes: Implications for diabetes management and patient education. Patient Educ Couns. 2007;68(1):10-5.
8. Kropff J, DeVries JH. Continuous Glucose Monitoring, Future Products, and Update on Worldwide Artificial Pancreas Projects. Diabetes Tech Ther. 2016;18 Suppl 2:S253-63.
9. Juvenile Diabetes Research Foundation Continuous Glucose Monitoring Study G, Tamborlane WV, Beck RW, Bode BW, Buckingham B, Chase HP, et al. Continuous glucose monitoring and intensive treatment of type 1 diabetes. N Engl J Med. 2008;359(14):1464-76.
10. Kordonouri O, Pankowska E, Rami B, Kapellen T, Coutant R, Hartmann R, et al. Sensor-augmented pump therapy from the diagnosis of childhood type 1 diabetes: results of the Paediatric Onset Study (ONSET) after 12 months of treatment. Diabetologia. 2010;53(12):2487-95.
11. Pickup JC, Freeman SC, Sutton AJ. Glycaemic control in type 1 diabetes during real time continuous glucose monitoring compared with self monitoring of blood glucose: meta-analysis of randomised controlled trials using individual patient data. Bmj. 2011;343:d3805.
12. Bergenstal RM, Tamborlane WV, Ahmann A, Buse JB, Dailey G, Davis SN, et al. Effectiveness of sensor-augmented insulin-pump therapy in type 1 diabetes. N Engl J Med. 2010;363(4):311-20.
13. Bergenstal RM, Klonoff DC, Garg SK, Bode BW, Meredith M, Slover RH, et al. Threshold-based insulin-pump interruption for reduction of hypoglycemia. N Engl J Med. 2013;369(3):224-32.
14. Thabit H, Hovorka R. Coming of age: the artificial pancreas for type 1 diabetes. Diabetologia. 2016;59(9):1795-805.
15. Hovorka R. Closed-loop insulin delivery: from bench to clinical practice. Nat Rev Endocrinol. 2011;7(7):385-95.
16. FDA News Release. FDA approves first automated insulin delivery device for type 1 diabetes. http://www.fda.gov/NewsEvents/Newsroom/PressAnnouncements/ucm522974.htm (last accessed 26 July 2018) [
17. Kropff J, Del Favero S, Place J, Toffanin C, Visentin R, Monaro M, et al. 2 month evening and night closed-loop glucose control in patients with type 1 diabetes under free-living conditions: a randomised crossover trial. Lancet Diabetes Endocrinol. 2015;3(12):939-47.
18. Hovorka R, Allen JM, Elleri D, Chassin LJ, Harris J, Xing D, et al. Manual closed-loop insulin delivery in children and adolescents with type 1 diabetes: a phase 2 randomised crossover trial. Lancet. 2010;375(9716):743-51.
19. Elleri D, Allen JM, Kumareswaran K, Leelarathna L, Nodale M, Caldwell K, et al. Closed-loop basal insulin delivery over 36 hours in adolescents with type 1 diabetes: randomized clinical trial. Diabetes Care. 2013;36(4):838-44.

For peer review only - http://bmjopen.bmj.com/site/about/guidelines.xhtml

20. Nimri R, Danne T, Kordonouri O, Atlas E, Bratina N, Biester T, et al. The "Glucositter" overnight automated closed loop system for type 1 diabetes: a randomized crossover trial. Pediatr Diabetes. 2013;14(3):159-67.
21. Hovorka R, Elleri D, Thabit H, Allen JM, Leelarathna L, El-Khairi R, et al. Overnight Closed Loop Insulin Delivery in Young People with Type 1 Diabetes: A Free-Living Randomised Clinical Trial. Diabetes Care. 2014;37(5):1204-11.
22. Thabit H, Tauschmann M, Allen JM, Leelarathna L, Hartnell S, Wilinska ME, et al. Home Use of an Artificial Beta Cell in Type 1 Diabetes. N Engl J Med. 2015;373(22):2129-40.
23. Nimri R, Muller I, Atlas E, Miller S, Kordonouri O, Bratina N, et al. Night glucose control with MD-Logic artificial pancreas in home setting: a single blind, randomized crossover trial-interim analysis. Pediatr Diabetes. 2014;15(2):91-9.
24. Tauschmann M, Allen JM, Wilinska ME, Thabit H, Stewart Z, Cheng P, et al. Day-and-Night Hybrid Closed-Loop Insulin Delivery in Adolescents With Type 1 Diabetes: A Free-Living, Randomized Clinical Trial. Diabetes Care. 2016;Jan 6 [Epub ahead of print].
25. Leelarathna L, Dellweg S, Mader JK, Allen JM, Benesch C, Doll W, et al. Day and night home closed-loop insulin delivery in adults with type 1 diabetes: three-center randomized crossover study. Diabetes Care. 2014;37(7):1931-7.
26. Thabit H, Lubina-Solomon A, Stadler M, Leelarathna L, Walkinshaw E, Pernet A, et al. Home use of closed-loop insulin delivery for overnight glucose control in adults with type 1 diabetes: a 4-week, multicentre, randomised crossover study. Lancet Diabetes Endocrinol. 2014;2(9):701-9.
27. Barnard KD, Wysocki T, Allen JM, Elleri D, Thabit H, Leelarathna L, et al. Closing the loop overnight at home setting: psychosocial impact for adolescents with type 1 diabetes and their parents. BMJ Open Diabetes Res Care. 2014;2(1):e000025.
28. Bekiari E, Kitsios K, Thabit H, Tauschmann M, Athanasiadou E, Karagiannis T, et al. Artificial pancreas treatment for outpatients with type 1 diabetes: systematic review and meta-analysis. Bmj. 2018;361:k1310.
29. Wilinska ME, Chassin LJ, Acerini CL, Allen JM, Dunger DB, Hovorka R. Simulation environment to evaluate closed-loop insulin delivery systems in type 1 diabetes. J Diabetes Sci Technol. 2010;4(1):132-44.
30. Boughton CK, Hovorka R. Is an artificial pancreas (closed-loop system) for Type 1 diabetes effective? Diabet Med. 2018.
31. Stevens K. Developing a descriptive system for a new preference-based measure of health-related quality of life for children. Qual Life Res. 2009;18(8):1105-13.
32. Wille N, Badia X, Bonsel G, Burstrom K, Cavrini G, Devlin N, et al. Development of the EQ-5D-Y: a child-friendly version of the EQ-5D. Qual Life Res. 2010;19(6):875-86.
33. Effectiveness of continuous glucose monitoring in a clinical care environment: evidence from the Juvenile Diabetes Research Foundation continuous glucose monitoring (JDRF-CGM) trial. Diabetes Care. 2010;33(1):17-22.
34. World Health Organization. Definition, Diagnosis and Classification of Diabetes Mellitus and its Complications. Part 1: Diagnosis and Classification of Diabetes Mellitus. WHO/NCD/NCS/99.2. Geneva, (1999).

For peer review only - http://bmjopen.bmj.com/site/about/guidelines.xhtml

Figure 1. Study flow chart

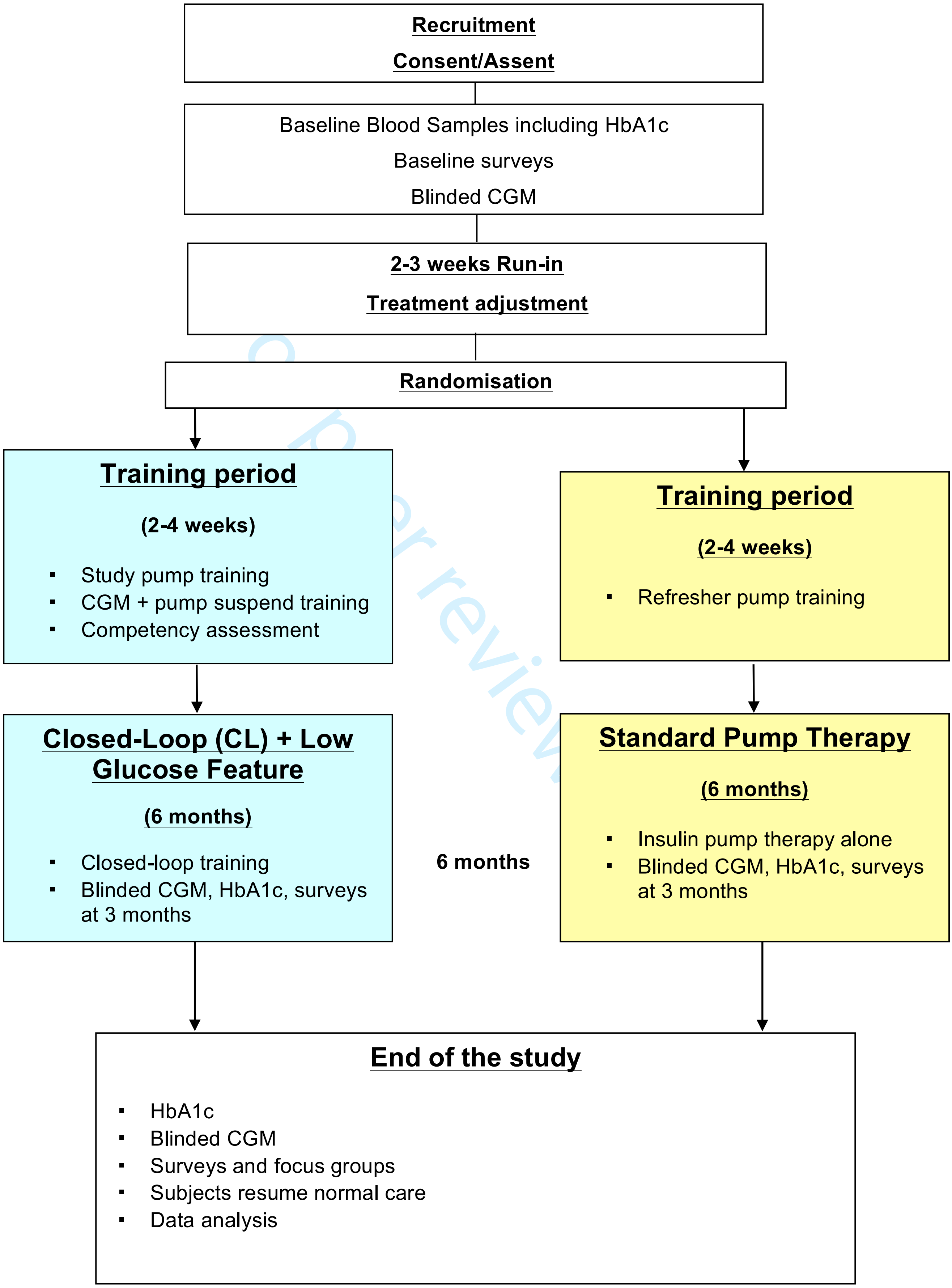

For peer review only - http://bmjopen.bmj.com/site/about/guidelines.xhtml

Figure 2. FlorenceM closed-loop system prototype

The system consists of a continuous glucose monitoring transmitter with Guardian 3 sensor (Medtronic), an insulin pump (modified 640G pump, Medtronic), and an Android smartphone running the control algorithm (Cambridge).

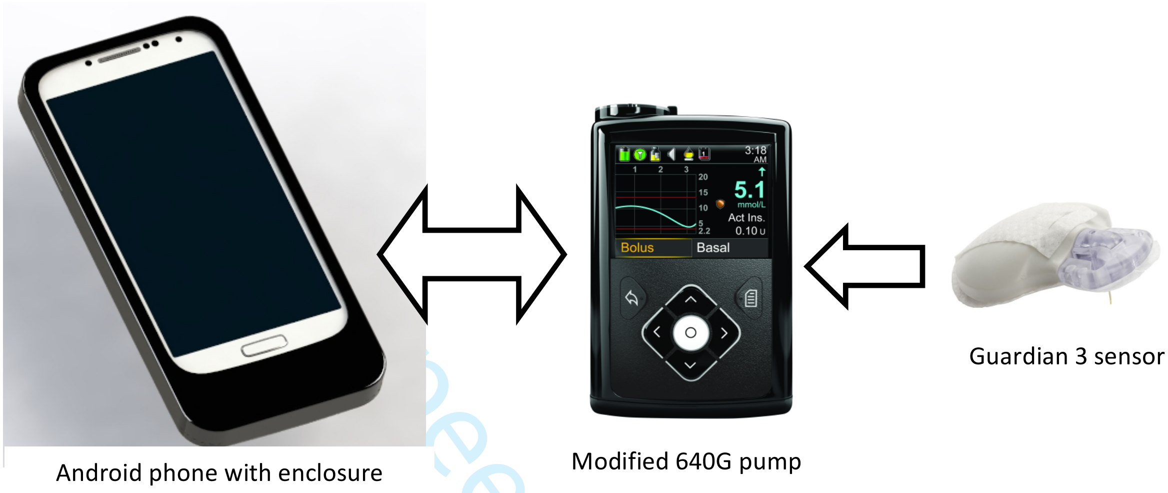

peer review only

For peer review only - http://bmjopen.bmj.com/site/about/guidelines.xhtml

Reporting checklist for protocol of a clinical trial.

Based on the SPIRIT guidelines.

Instructions to authors

Complete this checklist by entering the page numbers from your manuscript where readers will find each of the items listed below.

Your article may not currently address all the items on the checklist. Please modify your text to include the missing information. If you are certain that an item does not apply, please write "n/a" and provide a short explanation.

Upload your completed checklist as an extra file when you submit to a journal.

In your methods section, say that you used the SPIRIT reporting guidelines, and cite them as:

Chan A-W, Tetzlaff JM, Altman DG, Laupacis A, Gøtzsche PC, Krleža-Jerić K, Hróbjartsson A, Mann H, Dickersin K, Berlin J, Doré C, Parulekar W, Summerskill W, Groves T, Schulz K, Sox H, Rockhold FW, Rennie D, Moher D. SPIRIT 2013 Statement: Defining standard protocol items for clinical trials. Ann Intern Med. 2013;158(3):200-207

		Reporting Item	Page Number
Title	#1	Descriptive title identifying the study design, population, interventions, and, if applicable, trial acronym	1
Trial registration	#2a	Trial identifier and registry name. If not yet registered, name of intended registry	2

For peer review only

			NCT02925299 (ClinicalTrials.gov) DAN05
Trial registration: data set	#2b	All items from the World Health Organization Trial Registration Data Set	04/10/2016
Protocol version	#3	Date and version identifier	16.04.2018 (6.0)
Funding	#4	Sources and types of financial, material, and other support	14
Roles and responsibilities: contributorship	#5a	Names, affiliations, and roles of protocol contributors	14
Roles and responsibilities: sponsor contact information	#5b	Name and contact information for the trial sponsor	NCT02925299 (ClinicalTrials.gov)
Roles and responsibilities: sponsor and funder	#5c	Role of study sponsor and funders, if any, in study design; collection, management, analysis, and interpretation of data; writing of the report; and the decision to submit the report for publication, including whether they will have ultimate authority over any of these activities	NCT02925299 (ClinicalTrials.gov)

For peer review only

Roles and responsibilities: committees	#5d	Composition, roles, and responsibilities of the coordinating centre, steering committee, endpoint adjudication committee, data management team, and other individuals or groups overseeing the trial, if applicable (see Item 21a for data monitoring committee)	See supplementary file
Background and rationale	#6a	Description of research question and justification for undertaking the trial, including summary of relevant studies (published and unpublished) examining benefits and harms for each intervention	4
Background and rationale: choice of comparators	#6b	Explanation for choice of comparators	4
Objectives	#7	Specific objectives or hypotheses	2, 5
Trial design	#8	Description of trial design including type of trial (eg, parallel group, crossover, factorial, single group), allocation ratio, and framework (eg, superiority, equivalence, non-inferiority, exploratory)	5
Study setting	#9	Description of study settings (eg, community clinic, academic hospital) and list of countries where data will be collected. Reference to where list of study sites can be obtained	5

For peer review only

Eligibility criteria	#10	Inclusion and exclusion criteria for participants. If applicable, eligibility criteria for study centres and individuals who will perform the interventions (eg, surgeons, psychotherapists)	15
Interventions: description	#11a	Interventions for each group with sufficient detail to allow replication, including how and when they will be administered	5, 16, 17
Interventions: modifications	#11b	Criteria for discontinuing or modifying allocated interventions for a given trial participant (eg, drug dose change in response to harms, participant request, or improving / worsening disease)	See protocol
Interventions: adherence	#11c	Strategies to improve adherence to intervention protocols, and any procedures for monitoring adherence (eg, drug tablet return; laboratory tests)	N/A
Interventions: concomitant care	#11d	Relevant concomitant care and interventions that are permitted or prohibited during the trial	7
Outcomes	#12	Primary, secondary, and other outcomes, including the specific measurement variable (eg, systolic blood pressure), analysis metric (eg, change from baseline, final value, time to event), method of aggregation (eg, median, proportion), and time point for each outcome.	9, 10

For peer review only

		Explanation of the clinical relevance of chosen efficacy and harm outcomes is strongly recommended	
Participant timeline	#13	Time schedule of enrolment, interventions (including any run-ins and washouts), assessments, and visits for participants. A schematic diagram is highly recommended (see Figure)	See Figure 1
Sample size	#14	Estimated number of participants needed to achieve study objectives and how it was determined, including clinical and statistical assumptions supporting any sample size calculations	12
Recruitment	#15	Strategies for achieving adequate participant enrolment to reach target sample size	5
Allocation: sequence generation	#16a	Method of generating the allocation sequence (eg, computer-generated random numbers), and list of any factors for stratification. To reduce predictability of a random sequence, details of any planned restriction (eg, blocking) should be provided in a separate document that is unavailable to those who enrol participants or assign interventions	6

For peer review only

Allocation concealment mechanism	#16b	Mechanism of implementing the allocation sequence (eg, central telephone; sequentially numbered, opaque, sealed envelopes), describing any steps to conceal the sequence until interventions are assigned	6
Allocation: implementation	#16c	Who will generate the allocation sequence, who will enrol participants, and who will assign participants to interventions	
Blinding (masking)	#17a	Who will be blinded after assignment to interventions (eg, trial participants, care providers, outcome assessors, data analysts), and how	N/A
Blinding (masking): emergency unblinding	#17b	If blinded, circumstances under which unblinding is permissible, and procedure for revealing a participant's allocated intervention during the trial	N/A
Data collection plan	#18a	Plans for assessment and collection of outcome, baseline, and other trial data, including any related processes to promote data quality (eg, duplicate measurements, training of assessors) and a description of study instruments (eg, questionnaires, laboratory tests) along with their reliability and validity, if known. Reference to where data	See protocol

For peer review only

		collection forms can be found, if not in the protocol	
Data collection plan: retention	#18b	Plans to promote participant retention and complete follow-up, including list of any outcome data to be collected for participants who discontinue or deviate from intervention protocols	See protocol
Data management	#19	Plans for data entry, coding, security, and storage, including any related processes to promote data quality (eg, double data entry; range checks for data values). Reference to where details of data management procedures can be found, if not in the protocol	12
Statistics: outcomes	#20a	Statistical methods for analysing primary and secondary outcomes. Reference to where other details of the statistical analysis plan can be found, if not in the protocol	9
Statistics: additional analyses	#20b	Methods for any additional analyses (eg, subgroup and adjusted analyses)	11
Statistics: analysis population and missing data	#20c	Definition of analysis population relating to protocol non-adherence (eg, as randomised analysis), and any statistical methods to handle missing data (eg, multiple imputation)	11

For peer review only

Data monitoring: formal committee	#21a	Composition of data monitoring committee (DMC); summary of its role and reporting structure; statement of whether it is independent from the sponsor and competing interests; and reference to where further details about its charter can be found, if not in the protocol. Alternatively, an explanation of why a DMC is not needed	12
Data monitoring: interim analysis	#21b	Description of any interim analyses and stopping guidelines, including who will have access to these interim results and make the final decision to terminate the trial	11
Harms	#22	Plans for collecting, assessing, reporting, and managing solicited and spontaneously reported adverse events and other unintended effects of trial interventions or trial conduct	12-13
Auditing	#23	Frequency and procedures for auditing trial conduct, if any, and whether the process will be independent from investigators and the sponsor	12
Research ethics approval	#24	Plans for seeking research ethics committee / institutional review board (REC / IRB) approval	13
Protocol amendments	#25	Plans for communicating important protocol modifications (eg, changes to eligibility criteria,	13

For peer review only

		outcomes, analyses) to relevant parties (eg, investigators, REC / IRBs, trial participants, trial registries, journals, regulators)	
Consent or assent	#26a	Who will obtain informed consent or assent from potential trial participants or authorised surrogates, and how (see Item 32)	5
Consent or assent: ancillary studies	#26b	Additional consent provisions for collection and use of participant data and biological specimens in ancillary studies, if applicable	N/A
Confidentiality	#27	How personal information about potential and enrolled participants will be collected, shared, and maintained in order to protect confidentiality before, during, and after the trial	12
Declaration of interests	#28	Financial and other competing interests for principal investigators for the overall trial and each study site	14
Data access	#29	Statement of who will have access to the final trial dataset, and disclosure of contractual agreements that limit such access for investigators	12
Ancillary and post trial care	#30	Provisions, if any, for ancillary and post-trial care, and for compensation to those who suffer harm from trial participation	N/A

For peer review only

Dissemination policy: trial results	#31a	Plans for investigators and sponsor to communicate trial results to participants, healthcare professionals, the public, and other relevant groups (eg, via publication, reporting in results databases, or other data sharing arrangements), including any publication restrictions	9, 13
Dissemination policy: authorship	#31b	Authorship eligibility guidelines and any intended use of professional writers	See supplementary file
Dissemination policy: reproducible research	#31c	Plans, if any, for granting public access to the full protocol, participant-level dataset, and statistical code	
Informed consent materials	#32	Model consent form and other related documentation given to participants and authorised surrogates	Approved consents for UK and USA available
Biological specimens	#33	Plans for collection, laboratory evaluation, and storage of biological specimens for genetic or molecular analysis in the current trial and for future use in ancillary studies, if applicable	8

The SPIRIT checklist is distributed under the terms of the Creative Commons Attribution License CC-BY-ND 3.0. This checklist can be completed online using https://www.goodreports.org/, a tool made by the EQUATOR Network in collaboration with Penelope.ai

For peer review only

BMJ Open

Assessing the efficacy, safety and utility of 6 month day-and-night automated closed-loop insulin delivery under free living conditions compared to insulin pump therapy in children and adolescents with type 1 diabetes: an open-label, multi-centre, multi-national, single-period, randomised, parallel group study protocol

Journal:	*BMJ Open*
Manuscript ID	bmjopen-2018-027856.R1
Article Type:	Protocol
Date Submitted by the Author:	01-Mar-2019
Complete List of Authors:	Musolino, Gianluca; Wellcome Trust-MRC Institute of Metabolic Science, University of Cambridge; University of Cambridge, Department of Paediatrics<br>Allen, Janet; Wellcome Trust-MRC Institute of Metabolic Science, University of Cambridge ; University of Cambridge, Department of Paediatrics<br>Hartnell, Sara; Cambridge University Hospitals NHS Foundation Trust, Department of Diabetes and Endocrinology<br>Wilinska, Malgorzata; Wellcome Trust-MRC Institute of Metabolic Science, University of Cambridge ; University of Cambridge, Department of Paediatrics<br>Tauschmann, Martin; Wellcome Trust-MRC Institute of Metabolic Science, University of Cambridge; Medical University of Vienna, Department of Pediatrics and Adolescent Medicine<br>Boughton, Charlotte; Wellcome Trust-MRC Institute of Metabolic Science, University of Cambridge<br>Campbell, Fiona; Leeds Children's Hospital, Department of Paediatric Diabetes<br>Denvir, Louise; Nottingham University Hospitals NHS Trust, Department of Paediatric Diabetes and Endocrinology<br>Trevelyan, Nicola; Southampton Children's Hospital, Southampton General Hospital<br>Wadwa, Paul; Barbara Davis Center for Childhood Diabetes, University of Colorado<br>DiMeglio, Linda; Indiana University School of Medicine, Division of Pediatric Endocrinology and Diabetology and Wells Center for Pediatric Research, Department of Pediatrics<br>Buckingham, Bruce A.; Stanford University, Division of Pediatric Endocrinology<br>Weinzimer, Stuart; Yale University, Department of Pediatrics<br>Acerini, Carlo; Wellcome Trust-MRC Institute of Metabolic Science, University of Cambridge ; University of Cambridge, Department of Paediatrics<br>Hood, Korey; Stanford University, Division of Pediatric Endocrinology<br>Fox, Steven; University of Southern California, Department of

	Pharmaceutical and Health Economics, School of Pharmacy<br>Kollman, Craig; Jaeb Center for Health Research<br>Sibayan, Judy; Jaeb Center for Health Research<br>Borgman, Sarah; Jaeb Center for Health Research<br>Cheng, Peiyao; Jaeb Center for Health Research<br>Hovorka, Roman; Wellcome Trust-MRC Institute of Metabolic Science, University of Cambridge ; University of Cambridge, Department of Paediatrics

**Primary Subject Heading**:	Paediatrics
Secondary Subject Heading:	Diabetes and endocrinology
Keywords:	Type 1 diabetes, Closed-loop, Artificial pancreas

Assessing the efficacy, safety and utility of 6 month day-and-night automated closed-loop insulin delivery under free living conditions compared to insulin pump therapy in children and adolescents with type 1 diabetes: an open-label, multi-centre, multi-national, single-period, randomised, parallel group study protocol

[1,3]Musolino G, [1,3]Allen JM, [2]Hartnell S, [1,3]Wilinska ME, [1,4]Tauschmann M, [1]Boughton C, [5]Campbell F, [6]Denvir L, [7]Trevelyan N, [8]Wadwa RP, [9]DiMeglio LA, [10]Buckingham BA, [11]Weinzimer SA, [1,3]Acerini CL, [10]Hood KK, [12]Fox DS, [13]Kollman C, [13]Sibayan J, [13]Borgman S, [13]Cheng P, and [1,3]Hovorka R on behalf of DAN05 Consortium

[1]Wellcome Trust-MRC Institute of Metabolic Science, University of Cambridge, Cambridge, UK

[2]Department of Diabetes & Endocrinology, Cambridge University Hospitals NHS Foundation Trust, Cambridge, UK

[3]Department of Paediatrics, University of Cambridge, Cambridge, UK

[4]Department of Pediatrics and Adolescent Medicine, Medical University of Vienna, Austria

[5]Leeds Children's Hospital, Department of Paediatric Diabetes, Leeds, UK

[6]Department of Paediatric Diabetes and Endocrinology, Nottingham University Hospitals NHS Trust, Nottingham, UK

[7]Southampton Children's Hospital, Southampton General Hospital, Southampton, UK

[8]Barbara Davis Center for Childhood Diabetes, University of Colorado, Aurora, CO, USA

[9]Division of Pediatric Endocrinology and Diabetology and Wells Center for Pediatric Research, Department of Pediatrics, Indiana University School of Medicine, Indianapolis, IN, USA

[10]Division of Pediatric Endocrinology, Stanford University, Stanford, CA, USA

[11]Department of Pediatrics, Yale University, New Haven, CT, USA

[12]Department of Pharmaceutical and Health Economics, School of Pharmacy, University of Southern California, Los Angeles, CA, USA

[13]Jaeb Center for Health Research, Tampa, FL, USA

Correspondence

Roman Hovorka, PhD

University of Cambridge Metabolic Research Laboratories and NIHR Cambridge Biomedical Research Centre,

Wellcome Trust-MRC Institute of Metabolic Science, Box 289,

Addenbrooke's Hospital, Hills Road, Cambridge CB2 0QQ, UK

Tel: +44 1223 762 862, fax: +44 1223 330 598, e-mail: rh347@cam.ac.uk

Key Words: Type 1 diabetes, Closed-loop, Artificial pancreas

Word count abstract: 299 (maximum 300)

Word count paper: 3993 (maximum 4000)

For peer review only - http://bmjopen.bmj.com/site/about/guidelines.xhtml

ABSTRACT

Introduction

Closed-loop systems titrate insulin based on sensor glucose levels, providing novel means to reduce the risk of hypoglycaemia while improving glycaemic control. We will assess effectiveness of 6-month day-and-night closed-loop insulin delivery compared to usual care (conventional or sensor-augmented pump therapy) in children and adolescents with type 1 diabetes.

Methods and analysis

The trial adopts an open-label, multi-centre, multi-national (UK and USA), randomised, single-period, parallel design. Participants (n=130) are children and adolescents (age ≥ 6 and <19 years) with type 1 diabetes for at least 1 year, and insulin pump use for at least 3 months with sub-optimal glycaemic control [glycated haemoglobin ≥58mmol/mol (7.5%) and ≤86mmol/mol (10%)]. After a 2-3 week run-in period, participants will be randomised to 6-month use of hybrid closed-loop insulin delivery, or to usual care. Analyses will be conducted on an intention to treat basis. The primary outcome is glycated haemoglobin at 6 months. Other key endpoints include time in the target glucose range (3.9 to 10mmol/l, 70 to 180mg/dl), mean sensor glucose, and time spent above and below target. Secondary outcomes include standard deviation and coefficient of variation of sensor glucose levels, time with sensor glucose levels <3.5mmol/l (63mg/dl) and <3.0mmol/l (54mg/dl), area under the curve of glucose <3.5mmol/l (63mg/dl), time with glucose levels >16.7mmol/l (300mg/dl), area under the curve of glucose >10.0mmol/l (180mg/dl), total, basal and bolus insulin dose, body mass index z-score, and blood pressure. Cognitive, emotional and behavioural characteristics of participants and caregivers and their responses to the closed-loop and clinical trial will be assessed. An incremental cost-effectiveness ratio (ICER) for closed-loop will be estimated.

Ethics and dissemination

Cambridge South Research Ethics Committee and Jaeb Center for Health Research Institutional Review Office approved the study. The findings will be disseminated by peer-review publications and conference presentations.

Trial registration NCT02925299 (ClinicalTrials.gov).

For peer review only - http://bmjopen.bmj.com/site/about/guidelines.xhtml

Strengths and limitations of this study

- The study adopts an open-label, multi-centre, multi-national, randomised, parallel design: it includes a large group of children and adolescents across wide geographical locations
- The trial adopts a 6-month follow-up period of hybrid closed-loop insulin delivery during unrestricted living
- Participants in the two study groups will have an equal number of study visits
- The study design excludes participants with recurrent incidents of severe hypoglycaemia or diabetic ketoacidosis during the previous 6 months, living alone, and those with glycated haemoglobin below 58mmol/mol (7.5%) and above 86mmol/mol (10%) and with high or very low daily insulin requirements (total daily insulin dose ≥2IU/kg/day or <15IU/day)
- All participants are already pump users, somewhat limiting generalizability

For peer review only - http://bmjopen.bmj.com/site/about/guidelines.xhtml

INTRODUCTION

Type 1 diabetes is characterised by a deficiency of insulin caused by immunologically-mediated damage to pancreatic beta cells, leading to raised blood glucose levels. Diabetes is one of the most common metabolic conditions. It is estimated that in 2017 1,100,000 children and adolescents (0-19 years) worldwide had type 1 diabetes and that the number of newly diagnosed cases was over 130,000 (1). The incidence rate in children is increasing by approximately 3-4% per year with geographic differences (1). Earlier onset can result in diabetes complications appearing at a younger age, whilst dependence on lifelong insulin imposes a heavy burden on children, carers as well as health care systems.

Despite continuing progress, glycaemic control in children and adolescents with type 1 diabetes remains suboptimal (2). The achievement of recommended treatment goals is limited by the risk of hypoglycaemia. Even in those with the desired level of glycaemic control, non-physiological glucose excursions occur with periods of silent hyper- and hypoglycaemia (3, 4). Individuals have blunted counter-regulatory responses to hypoglycaemia impairing recovery and increasing the threat of future episodes (5). Recurrent episodes may lead to hypoglycaemic unawareness, increasing the risk of severe hypoglycaemia (6). Hypoglycaemia has psychological consequences including the fear of hypoglycaemia with resulting maladaptive coping behaviours, such as excessive eating or under-insulinising, that may negatively impact glycaemic control (7).

The development of continuous glucose monitoring has been a major advance (8-11). Sensor-augmented pumps combine real-time continuous glucose monitoring with insulin pump (12). Insulin pumps with low glucose suspend feature have been shown to reduce hypoglycaemia (13). These systems, however, overall provide little or no automation to adjust insulin delivery to match glucose excursions.

An artificial pancreas (a closed-loop system) adjusts insulin automatically and represents a realistic treatment option for type 1 diabetes (14). The closed-loop control algorithm translates, in real-time, sensor glucose levels received from the glucose monitoring device and computes the amount of insulin to be delivered by the coupled insulin pump. Hybrid closed-loop systems automatically titrate insulin delivery although the user manages insulin boosts at meal time (15). In 2017, the first closed-loop system entered clinical use in the USA (16).

Closed-loop systems may improve glycaemic control while reducing the risk of hypoglycaemia (17). They have been evaluated in children and adolescents under controlled laboratory conditions (18-20) and in home settings (21-24). Investigations in adults have also been conducted (22, 25, 26). Psychosocial assessments support acceptability and benefits of this therapeutic approach among children/adolescents and carers (27). Closed-loop systems are associated with increased time in near normoglycaemia and reduced time in hypoglycaemia and hyperglycaemia (28). So far, evaluations have been limited to 3 months (22).

The present study will assess the efficacy, safety, utility and acceptability of 6-month day-and-night hybrid closed-loop insulin delivery during unrestricted living in comparison to usual care in children and adolescents with type 1 diabetes.

For peer review only - http://bmjopen.bmj.com/site/about/guidelines.xhtml

METHODS AND ANALYSIS

Overview

This trial adopts an open-label, multi-centre, multi-national, single-period, randomised, parallel group design, involving a 6-month home study period during which day-and-night glucose levels will be managed either by a closed-loop system (intervention group) or by insulin pump therapy (control group) (Figure 1). We aim to recruit up to 150 children and adolescents aged ≥6 to <19 years with type 1 diabetes on insulin pump therapy (approximately equal proportion of those aged ≥6 to 12 years and 13 to <19 years, a minimum quota of 25% participants with baseline glycated haemoglobin >69mmol/mol, >8.5%). Inclusion and exclusion criteria are summarised in Table 1.

The University of Cambridge (UK) and Jaeb Center for Health Research (USA) are the coordinating centres. Clinical centres include:

1) Addenbrooke's Hospital, Cambridge, UK
2) Barbara Davis Center for Childhood Diabetes, Aurora, USA
3) Indiana University, Indianapolis, USA
4) Leeds Teaching Hospital, Leeds, UK
5) Nottingham Children's Hospital, Nottingham, UK
6) Southampton Children's Hospital, Southampton, UK
7) Stanford University, Stanford, California, USA
8) Yale University, New Haven, Connecticut, USA

Cognitive, emotional, and behavioural characteristics of participants and family members and their response to the closed-loop will be assessed gathering both quantitative (validated surveys) and qualitative data (focus groups). Written informed consent/assent will be obtained from all participants and guardians before any study-related activities.

Study schedule

The study will comprise up to 8 visits and 6 telephone/email contacts (see Table 2 and Table 3). The maximum study duration is 8 months.

Screening and baseline assessment

At screening, blood samples for full blood count, liver, thyroid function and anti-transglutaminase antibodies (with IgA levels if not done within previous 12 months) will be taken. Non-hypoglycaemia C-peptide, glucose and glycated haemoglobin will be measured and a urine pregnancy test in females of child-bearing potential will be performed. Surveys investigating participants' quality of life, psychosocial and cognitive functioning, and response to their current treatment will be distributed. Participants will be fitted with a blinded continuous glucose monitoring device (Libre Pro, Abbott Diabetes Care, Alameda, CA, USA) that will be worn during the run-in period at home for up to 14 days.

Run-in period

During a 2-3 week run-in period, subjects will continue using their own insulin pump. Data obtained from blinded glucose sensors and pump downloads may be utilised for treatment

For peer review only - http://bmjopen.bmj.com/site/about/guidelines.xhtml

adjustments. The run-in period may be extended/repeated if no or limited sensor data is available. At least 10 days of sensor data need to be collected.

Randomisation

Central randomisation software will be used with stratification by site and baseline glycated haemoglobin. The randomisation ratio will be 1:1 within each stratum. The randomisation list created by the study statistician is encrypted.

Treatment period

1. Automated day-and-night hybrid closed-loop insulin delivery combined with low glucose suspend feature (interventional arm)

Participants allocated to the closed-loop group will be trained on using the study insulin pump (modified Medtronic 640G pump, Medtronic, Northridge, CA, USA) and real-time continuous glucose sensor (Guardian 3, Medtronic). This represents a complex intervention over usual care, especially for subjects under pump therapy alone. Once deemed competent with the use of the devices, participants will receive training required for the closed-loop system. Competency on the use of closed-loop will be evaluated. During closed-loop period, participants will program meal boluses estimating ingested carbohydrate amounts. Specific instructions during closed-loop related to exercise management, sick day rules, hypo- and hyperglycaemia management and technical troubleshooting will be provided.

2. Usual care (conventional or sensor-augmented pump therapy) (control arm)

Participants in control arm will receive refresher training on key aspects of insulin pump therapy (advanced boluses, temporary basal, infusion set change, sensor calibrations). During 6-month control intervention period, subjects will continue using either their own insulin pump alone or combined with their pre-study glucose monitoring device.

At the study initiation visit, participants in both study groups will be fitted with a blinded continuous glucose monitoring system (Libre Pro) that will be worn for up to 14 days. If the sensor fails or gets detached, another sensor may be inserted. The sensor data may be used to optimise insulin delivery.

Assessments at 3 months and 6 months

A blood sample will be collected for measurement of glycated haemoglobin. A urine pregnancy test in females of child-bearing potential will be performed. As per usual clinical practice, glucometer downloads and pump data will be reviewed, and adjustments to insulin pump settings will be made as required. Validated surveys evaluating the impact of the devices employed on quality of life, psychosocial and cognitive functioning, diabetes management and treatment satisfaction will be administered. At the 3-month follow-up visit, participants in both study groups will be fitted with blinded continuous glucose monitoring systems (Libre Pro). For assessment of glycaemic control during the final 3-month period of the trial, participants in both study groups will be fitted with a blinded continuous glucose monitoring system 2 to 4 weeks before the end of study. At the 6-month visit, the same procedures as at the 3-month visit will be followed. A subset of subjects/guardians will be invited to join follow-up focus groups.

For peer review only - http://bmjopen.bmj.com/site/about/guidelines.xhtml

Study contacts during 6-month study period

Participants in the two study groups will have an equal number of contact visits. The first planned contact will occur within 24-48 hours after study initiation visit. During the first 2 weeks of the study period, participants will be contacted weekly. Thereafter, participants will be contacted monthly. Subjects/parents and/or the clinical team are free to adjust insulin therapy, but no active treatment optimisation will be undertaken by the research team.

Devices download

As per usual care, insulin pump and blood glucose meter will be downloaded (Medtronic CareLink) every clinic visit (at least every 3 months).

Closed-loop system

The FlorenceM closed-loop system (Figure 2) incorporates a computer-based algorithm hosted by an Android smartphone, which interacts wirelessly with the modified investigational-use-only 640G pump through a proprietary translator device included in the smartphone's enclosure. By using the information received from the glucose sensor, every ten minutes the system computes a new temporary basal insulin infusion rate, which is automatically sent to the insulin pump. The treat-to-target control algorithm aims to achieve a default glucose level of 5.8mmol/l (104mg/dl) and regulates the actual level depending on fasting versus postprandial status and the accuracy of model-based glucose predictions. No remote monitoring is planned. While the system is charging and connected to internet, the device uploads data on a server. The study pump comprises continuous glucose monitoring receiver and provides hypoglycaemia and hyperglycaemia alarms, which can be activated and personalised by the participants.

Safety precautions during closed-loop

Participants will be asked to perform capillary calibrations before breakfast and dinner. If sensor glucose value is >3.0mmol/l (54mg/dl) different from capillary glucose level, the sensor will be recalibrated. These directions are based on an in-silico simulation of hypo- and hyperglycaemia risk using the validated Cambridge simulator (29). If sensor glucose becomes unavailable or the smartphone is not in range/operational, the pump will automatically deliver the pre-programmed insulin within 30 minutes. Safety rules limit maximum insulin infusion and suspend insulin delivery when sensor glucose is ≤4.3mmol/l (77mg/dl) or when glucose is rapidly decreasing. In case of a communication failure between control algorithm device and the study pump, the low-glucose feature will interrupt insulin delivery, provided sensor glucose is available. Insulin delivery will be resumed in accordance of the low glucose suspend feature implemented on the study pump. A 24-hour local telephone helpline will be available for any technical device issues or problems related to diabetes management.

Participant withdrawal criteria

The following pre-randomisation withdrawal criteria will apply:

1. Subject/caregiver is unable to demonstrate safe use of study insulin pump as judged by the investigator

For peer review only - http://bmjopen.bmj.com/site/about/guidelines.xhtml

2. Subject/caregiver fails to demonstrate compliance with insulin pump and capillary self-monitoring of blood glucose during run-in

Pre- and post-randomisation withdrawal criteria will comprise:

3. Subjects/caregivers may terminate participation in the study at any time without necessarily giving a reason and without any personal disadvantage
4. Significant protocol violation or non-compliance
5. Two distinct episodes of severe hypoglycaemia
6. Two distinct episodes of diabetic ketoacidosis unrelated to infusion site failure and related to the use of the closed-loop
7. Decision by the investigator or the sponsor that termination is in the subject's best medical interest
8. Allergic reaction to insulin
9. Allergic reaction to adhesive surface of infusion set or glucose sensor
10. Subject becomes pregnant during the study period

Subjects withdrawn due to reasons 4-10 will be invited to provide blood sample at the end of the planned study intervention for the assessment of glycated haemoglobin.

Psychosocial evaluations

Cognitive, emotional, and behavioural characteristics of participating subjects and family members and their response to the closed-loop system and clinical trial will be assessed using validated surveys and focus groups. Surveys will be completed at baseline (prior to randomisation), at 3, and 6 months.

To assess how strongly participants value the benefits of the closed-loop (compared with the usual care), we will conduct a discrete choice experiment (DCE). In the DCE, respondents will answer a series of binary choice questions (e.g., "Given a choice between option A or B, which would you prefer...") where those two options offer differing strengths and weaknesses. By varying the performance levels of these different desirable characteristics, we can assess their relative importance.

Focus groups will be completed at the end of the study (6 months). We will conduct virtual focus groups using HIPAA-approved software supported by Stanford University. Focus groups will be run with 3-6 participants and we will work from a script of open-ended questions used to gather feedback and reactions to the closed-loop system/insulin pump therapy, the clinical trial and quality of life changes. The participation of a moderator with advanced training will ensure consistency across groups. Sessions will be audio- and video-taped and transcribed by a professional transcription service.

Blood samples

Screening blood samples will be measured locally. Additional blood samples will be taken for the measurement of non-hypoglycaemia C-peptide and glycated haemoglobin at a central laboratory. Glycated haemoglobin will be assessed at baseline, 3, and 6 months. At each time point, glycated haemoglobin will be measured locally (clinical care) and centrally (analysis of study endpoints). The central analysis will be performed using an International Federation of Clinical Chemistry and Laboratory Medicine aligned method.

For peer review only - http://bmjopen.bmj.com/site/about/guidelines.xhtml

Patient and Public Involvement

The research question and study endpoints are based on feedback from participants of previous studies and in line with prioritising by stakeholders (30). The study design and the assessment of the burden of the intervention were reviewed by focus groups. Results will be disseminated to participants and general public through social media and will be made available on the sponsor's website.

Statistical analysis

Primary Outcome Analysis

The primary analysis will follow the intention-to-treat principle. Data from all randomised subjects will be analysed in the group to which the subjects were assigned through randomisation regardless of the actual treatment received. Data will not be truncated due to protocol deviations.

The primary analysis will evaluate between group differences in glycated haemoglobin levels at the end of treatment period. A 5% significance level will be considered statistically significant for the primary outcome comparison.

Means ± standard deviation (SD) values or percentiles appropriate to the distribution will be reported for the primary outcome by treatment group. The two treatment groups will be compared using a linear regression model adjusting for glycated haemoglobin at baseline, age, and clinical centre as random effect. A 95% confidence interval will be reported for the difference between the randomisation groups based on the linear regression model. Residual values will be examined for an approximate normal distribution. If values are highly skewed, then a transformation or robust statistical methods (e.g., non-parametric or MM estimation) will be used instead. A detailed analysis plan will be provided separately.

Other Key Endpoints

For the following key endpoints at 6 months, the familywise type I error rate will be controlled at two-sided $\alpha = 0.05$. A gatekeeping strategy will be used, where the primary endpoint will be tested first, if passing the significance testing, other key endpoints will be tested in the order listed below using the fixed-sequence method at $\alpha = 0.05$.

- Time spent in the target glucose range from 3.9 to 10.0mmol/l (70 to 180mg/dl)
- Mean sensor glucose
- Time spent above target glucose 10.0mmol/l (180mg/dl)
- Time spent below target glucose 3.9mmol/l (70mg/dl)

If a non-significant ($p > 0.05$) result is obtained for any outcome on this list, no further hypothesis testing will be performed for any metrics further down on the list.

Secondary Efficacy Analyses

For these exploratory analyses, the false discovery rate will be used to account for multiple comparisons:

<u>Continuous glucose monitoring derived indices</u>

- Standard deviation of sensor glucose
- Sensor glucose variability measured with the coefficient of variation
- The time with glucose <3.5mmol/l (63mg/dl)

- The time with glucose <3.0mmol/l (54mg/dl)
- Area under the curve of glucose below 3.5mmol/l (63mg/dl)
- The time spent in significant hyperglycaemia (glucose >16.7mmol/l, 300mg/dl)
- Area under the curve of glucose above 10.0mmol/l (180mg/dl)

The following sensor glucose metrics will also be calculated separately for day-time period (06:00-23:59) and night-time period (00:00-05:59):

- The time with glucose from 3.9 to 10.0mmol/l (70-180mg/dl)
- Mean glucose
- Glucose variability as measured by standard deviation
- The time with glucose <3.5mmol/l (63mg/dl)

Binary metrics for glycated haemoglobin

- HbA1c <53mmol/mol (7.0%)
- HbA1c <58mmol/mol (7.5%)
- Relative reduction ≥10% from baseline
- Absolute reduction ≥0.5% from baseline
- Absolute reduction ≥1% from baseline
- Absolute reduction ≥1% from baseline or HbA1c <53mmol/mol (7.0%)

Insulin and other endpoints

- Total, basal and bolus insulin dose
- Body weight (BMI z-score)
- Blood pressure

The above described glycaemic metrics will be based on sensor glucose levels collected during post-randomisation periods of blinded sensors wear.

Safety analyses

The following events will be recorded and compared between treatment groups:

- Number of severe hypoglycaemia events per subject and incidence rate per 100 person-years
- Number of diabetic ketoacidosis events per subject and incidence rate per 100 person-years
- Sensor glucose-measured hypoglycaemic events per week (>15 minutes with glucose <3mmol/l, 54mg/dl)
- Sensor glucose-measured hyperglycaemic events per week (>15 minutes with glucose >16.7mmol/l, 300mg/dl)
- Proportion of subjects with worsening of glycated haemoglobin from baseline to 6 months by >0.5%

If we record enough observed events to allow formal statistical modelling for above safety outcomes, we will perform the following analyses. Poisson regression models will be constructed to compare the treatment group difference for event rates by adjusting for age, baseline glycated haemoglobin and random site effect. If any outlier exists, a robust Poisson regression model will be used instead. For binary glycated haemoglobin outcome, logistic

regression models will be used to compare the treatment group difference by adjusting for age, baseline glycated haemoglobin and random site effect.

Utility assessments

The following system use/function outcomes in the intervention arm will be tabulated:

- Number of low glucose suspend events
- Percentage of time when closed-loop system use is functioning
- Percentage of time when continuous glucose monitoring is used

Subgroup analyses

No subgroups were considered during the power calculations. Interpretation of any subgroup analyses will depend on whether the overall analysis demonstrates a significant treatment group difference. In the absence of such difference, if performed, the subgroup analyses will be interpreted with caution.

Psychosocial analyses

Quantitative data on usability and satisfaction will be analysed using simple descriptive statistics. Additionally, we will analyse scores from the cognitive, emotional, and behavioural assessments to determine if changes occur over time and between groups.

We will construct predictive models in the general linear framework to examine the associations with primary outcomes. For the discrete choice experiment (DCE), the strength of preference (importance) of each performance attribute will be estimated from the pooled DCE responses using standard regression analysis techniques.

Qualitative data will be analysed using Atlas.ti (release 6.0; Scientific Software Development GmbH, Berlin, Germany) to organize and manage the entire corpus of focus group data.

Cost utility analyses

To inform reimbursement and other policy decision-making, we will conduct a cost utility analysis on the benefits of closed-loop. The analysis timeframe for both costs and benefits will include not just the study period, but also anticipated future impacts. Costs will be denominated in US Dollars. They will be framed to include both health-related expenditures and any realised or projected incremental health cost savings. Utility will be quantified in quality adjusted life years (QALYS). We will elicit health related quality of life (HRQOL) during the study period using two preference based measures of health status: the Child Health Utility 9D (31) and the EuroQol 5D-Y (32). Future health and cost impacts, beyond the study period, will be estimated using numerical modelling. Incremental cost effectiveness ratios, comparing the closed-loop system to usual care will be calculated.

Interim analysis

We will not perform an interim analysis.

For peer review only - http://bmjopen.bmj.com/site/about/guidelines.xhtml

Per-protocol analysis

We will conduct a per-protocol analysis in order to replicate the primary analysis, but limited to participants who did not withdraw from the study (withdrawals excluded even if they return for a 6-month glycated haemoglobin measurement) and used closed-loop for at least 70% of the time (intervention group).

Power calculation

Data from the JDRF Continuous Glucose Monitoring Randomised Clinical Trial (33) from subjects who would have met the eligibility criteria for the current trial were used to project the distribution of baseline and 6-month glycated haemoglobin. Among N=53 subjects meeting the eligibility criteria in the JDRF CGM RCT (n=20 subjects 8 to 12 years of age and n=33 subjects 13 to 18 years of age), the upper limit of the confidence interval for the effective SD of glycated haemoglobin was 0.71%. With this effective SD, for a true 0.4% reduction in glycated haemoglobin, power = 85%, 2-sided type 1 error = 5%, 1:1 randomisation, total sample size is estimated to be 116. Adding 10% for potential dropout/non-compliance results in a final total sample size of approximately 128 (64 in each treatment group).

STUDY MANAGEMENT

Data Safety Monitoring Board

A Data Safety Monitoring Board (DSMB) will be instituted. The DSMB will be notified of all serious adverse events and any unanticipated adverse device effects/events and will perform regular safety data review. The DSMB will report to the National Institute of Diabetes and Digestive and Kidney Diseases (the Funder) any safety concerns and recommendations for suspension or early termination of the trial.

Study sponsors

In the UK the study sponsors are the University of Cambridge and the Cambridge University Hospitals NHS Foundation Trust. Study sponsor in the USA is the Jaeb Center for Health Research.

Study management committee

A study management committee composed of the Chief Investigator, Study Coordinators, and Study Data Manager will meet monthly to discuss the operational aspects of the trial.

Data management and monitoring

Designated personnel from Coordinating Centres will be responsible for maintaining quality assurance and quality control systems to ensure that the trial is conducted and data are generated, documented and reported in compliance with the protocol, Good Clinical Practice, and regulatory requirements.

We will observe confidentiality of subject data. Personal details for each participant with a link to a unique identification number will be held locally on a study screening log in the Trial Master File at each of the investigation centres. These details will not be disclosed at any other stage during the study, and all individual results will remain anonymous.

Indemnity

Indemnity for any harm rising on the conduct of research will be provided according to arrangements in respective countries:

1) UK - any liability arising from study design will be covered by clinical trial insurance policy organised by the University of Cambridge. National Health Service indemnity cover will apply for any claims arising from management and conduct of research.

2) USA - any liability arising from study design will be under the responsibility of the participants or their insurance company.

ETHICS AND DISSEMINATION

Approval from independent Research Ethics Committee/Institutional Review Board has been obtained in the UK and the USA. The study has undergone a review by regulatory authorities in the UK (Medicines and Healthcare products Regulatory Agency) and in the USA (Food and Drug Administration). All participants will be provided with oral and written information about the trial and procedures involved in the study before obtaining written informed consent. For minors, parents/guardians will provide written informed consent, and written assent will be gained.

Standard operating procedures for monitoring and reporting of all adverse events and adverse device effects will be in place including serious adverse events, serious adverse device effects and specific adverse events, such as severe hypoglycaemia and significant hyperglycaemia with ketosis.

Any substantial amendments to the protocol and other documents shall be submitted to, and approved by, the independent Research Ethics Committee and Institutional Review Board (UK, East of England-Cambridge South Research Ethics Committee, #16/EE/0380; USA, Jaeb Center for Health Research Institutional Review Board certified by the Office for Human Research Protections, FWA #00000024) and the regulatory authorities, prior to implementation as per nationally agreed guidelines.

The study started enrolling participants in June 2017 and is expected to complete clinical follow up by November 2019 and to report results in 2020. Trial results will be disseminated in internationally peer-reviewed scientific journals.

For peer review only - http://bmjopen.bmj.com/site/about/guidelines.xhtml

Acknowledgements
Jasdip Mangat supported development and validation of closed-loop system. Josephine Hayes, Matthew Haydock and Nicole Ashcroft (Institute of Metabolic Science, University of Cambridge) provided administrative support. NIHR Cambridge Clinical Research Facility will support the research team in their research-related activities. Artificial Pancreas focus group contributors provided feedback on the study design. West Midlands Young Persons Advisory Group reviewed Participant Information Sheets.

Contributors
RH, MEW, FC, LD, NT, RPW, LADM, BAB, SAW, CLA, and KKH co-designed the study. CK and PC designed the statistical plan. GM, JMA, SH, MT, CB, FC, LD, NT, RPW, LADM, BAB, SAW, and CLA screened and enrolled participants, arranged informed consent from the participants, provided patient care, and took samples. KKH devised the human factors assessments. JS, SB coordinated the study. JS managed randomisation. DSF will conduct the cost utility analysis. RH designed and implemented the glucose controller. GM and RH wrote the manuscript. All authors critically reviewed the report. No writing assistance was provided.

Funding
National Institute of Diabetes and Digestive and Kidney Diseases of the National Institutes of Health under Award Number UC4DK108520. Additional support for the artificial pancreas work is from National Institute for Health Research Cambridge Biomedical Research Centre, and Wellcome Strategic Award (100574/Z/12/Z). Medtronic is supplying discounted CGM devices, sensors, and details of communication protocol to facilitate real-time connectivity. Abbott Diabetes Care is providing Libre Pro sensors.

Relevant disclosures
RH reports having received speaker honoraria from Eli Lilly and Novo Nordisk, serving on advisory panel for Eli Lilly and Novo Nordisk, receiving licence fees from BBraun and Medtronic. RH and MEW report patent patents and patent applications.
MT has received speaker honoraria from Medtronic and NovoNordisk.
RPW reports receiving speaker honoraria from Dexcom and serving on advisory panels for Eli Lilly and Novo Nordisk and research support from Bigfoot Biomedical, Dexcom, Lexicon, Mannkind and Novo Nordisk. BAB is on Advisory Boards for Novo-Nordisk and Convatec, has received research support from Medtronic Diabetes, Tandem Diabetes, Insulet, Convatec, and Dexcom. SAW has received speaker honoraria from Medtronic, Insulet, and Tandem, and has received consultant honoraria from Sanofi and Zealand Pharmaceuticals.
KKH has received research support from Dexcom, Inc for an investigator-initiated project; he has received consultant fees from Lilly Innovation Center, Bigfoot Biomedical, and Insulet, Inc. LADM reports grants from Medtronic.
GM, JMA, SH, CB, FC, LD, NT, CLA, DSF, CK, JS, SB, PC declare no competing financial interests exist.

Ethics and IRB approvals
East of England - Cambridge South Research Ethics Committee (UK), Jaeb Center for Health Research Institutional Review (IRB) Office (813-975-8690 or irb@jaeb.org) (USA).

Provenance and peer review
Not commissioned, internally peer reviewed.

Table 1. Inclusion and exclusion criteria

**Summary of inclusion criteria**
▪ Age ≥6 and <19 years<br>▪ Type 1 diabetes as defined by World Health Organization (34) for at least 1 year<br>▪ Use of an insulin pump for at least 3 months, with good knowledge of insulin self-adjustment by subject or caregiver as judged by the investigator<br>▪ Using U-100 rapid acting insulin analogues Aspart or Lispro only<br>▪ Willing to perform regular finger-prick blood glucose monitoring, with at least 4 blood glucose measurements per day<br>▪ Screening glycated haemoglobin ≥58 mmol/mol (7.5%) and ≤86mmol/mol (10%) based on analysis from local laboratory<br>▪ Literate in English<br>▪ Willing to wear continuous glucose sensor and closed-loop system at home<br>▪ Willing to follow study specific instructions<br>▪ Willing to upload pump and glucose sensor data at regular intervals<br>▪ Access to Wi-Fi<br>▪ Living with someone who is trained to administer glucagon and is able to seek emergency assistance
**Summary of exclusion criteria**
▪ Living alone<br>▪ Current use of any closed-loop system<br>▪ Any other physical or psychological disease likely to interfere with the normal conduct of the study and interpretation of the study results, as judged by the investigator<br>▪ Untreated coeliac disease, adrenal insufficiency, or untreated thyroid disease<br>▪ Current treatment with drugs known to interfere with glucose metabolism (e.g., systemic corticosteroids, non-selective beta-blockers and monoamine oxidase inhibitors, etc.)<br>▪ Known or suspected allergy to insulin<br>▪ Clinically significant nephropathy (estimated glomerular filtration rate <45ml/min) or on dialysis, neuropathy or active retinopathy (presence of maculopathy or proliferative changes), as judged by the investigator<br>▪ Recurrent incidents of severe hypoglycaemia (>1 episode) during the previous 6 months (adolescents: severe hypoglycaemia is defined as an event requiring assistance of another person to actively administer carbohydrates, glucagon, or take other corrective actions including episodes of hypoglycaemia severe enough to cause unconsciousness, seizures or attendance at hospital; children: severe hypoglycaemia is defined as an event associated with a seizure or loss of consciousness)<br>▪ Recurrent incidents of diabetic ketoacidosis (>1 episode) during the previous 6 months<br>▪ Unwilling to avoid regular use of acetaminophen<br>▪ Lack of reliable telephone facility for contact<br>▪ Total daily insulin dose ≥2 IU/kg/day and <15 IU/day<br>▪ Pregnancy, planned pregnancy, or breast feeding<br>▪ Severe visual or hearing impairment<br>▪ Seizure disorder<br>▪ Medically documented allergy towards the adhesive (glue) of plasters or unable to tolerate tape adhesive in the area of sensor placement<br>▪ Serious skin diseases (e.g., psoriasis vulgaris, bacterial skin diseases) located at places of the body likely to be used for localisation of the glucose sensor<br>▪ Abusing illicit drugs, prescription drugs or alcohol<br>▪ Use of pramlintide (Symlin), or other non-insulin glucose lowering agents including sulphonylureas, biguanides, DPP4-Inhibitors, GLP-1 analogues, SGLT-1/ 2 inhibitors at time of screening<br>▪ Shift work with working hours between 10pm and 8am<br>▪ Sickle cell disease, haemoglobinopathy, or has received red blood cell transfusion or erythropoietin within 3 months prior to time of screening<br>▪ Eating disorder such as anorexia or bulimia<br>▪ Employed by Medtronic Diabetes or with immediate family members employed by Medtronic Diabetes

Table 2. Schedule of study visits / phone contacts when the participant is randomised to day-and-night closed-loop combined with low glucose feature (intervention group)

	Visit/ contact	Description	Start relative to previous / next Visit / Activity	Duration
**Run-in**	**Visit 1**	Recruitment visit: consent, HbA1c, screening bloods, urine pregnancy test, baseline surveys, blinded CGM training and insertion		1-4 hours
	**Visit 2**	Review of baseline bloods, pump settings and CGM data; adjustment of treatment	2 weeks after Visit 1 (+1 week); Run-in could be repeated	1-2 hours
**Training Period**	**Visit 3**	Randomisation, repeat HbA1c if Visit 3 and Visit 1 are >28 days apart, urine pregnancy test, study pump training and initiation, competency assessment	May coincide with Visit 2, within 8 weeks of Visit 1	3-4 hours
	**Visit 3a**	Real-time CGM training and initiation, competency assessment	Within 0 to 7 days of Visit 3 (Visit 3a may coincide with Visit 3; training visits can be repeated)	2-4 hours
**CL + LGS Intervention (6 months)**	**Visit 4***	CL initiation at clinic/home: data download, CL and low glucose feature training, competency assessment, blinded CGM	4 weeks after Randomisation (±1 week)	2-6 hours
	Contact 1	Review use of study devices; study update	Within 24 to 48 hours after Visit 4	<1 hour
	**Visit 5****	Review use of study devices; study update	1 week after Visit 4 (± 3 days)	<1 hour
	Contact 2	Review use of study devices; study update	2 weeks after Visit 4 (±3 days)	<1 hour
	Contact 3	Review use of study devices; study update	1 month after Visit 4 (±2 weeks)	<1 hour
	Contact 4	Review use of study devices; study update	2 months after Visit 4 (±2 weeks)	<1 hour
	**Visit 6**	3-month visit: HbA1c, urine pregnancy test, data download, blinded CGM, surveys	4 months after Randomisation (±2 weeks)	1-3 hours
	Contact 5	Review use of study devices; study update	5 months after Randomisation (±2 weeks)	<1 hour
	Contact 6	Review use of study devices; study update	6 months after Randomisation (±2 weeks)	<1 hour
	**Visit 7**	Blinded CGM	2-4 weeks before planned Visit 8	<0.5 hour
	**Visit 8**	End of closed-loop treatment arm (6 months of CL): HbA1c, data download, surveys and focus groups; resume usual pump therapy	7 months after Randomisation (±2 weeks)	1-3 hours

* In-person clinic visit mandatory in USA only.
** Could be done via phone/e-mail in UK. In-person visit mandatory in USA only.
HbA1c, glycated haemoglobin; CGM, continuous glucose monitoring; CL, closed-loop.

For peer review only - http://bmjopen.bmj.com/site/about/guidelines.xhtml

Table 3. Schedule of study visits / phone contacts when the participant is randomised to usual care (conventional or sensor-augmented insulin pump therapy) (control group)

	Visit/ contact	Description	Start relative to previous / next Visit / Activity	Duration
**Run-in**	**Visit 1**	Recruitment visit: consent, HbA1c, screening bloods, urine pregnancy test, baseline surveys, blinded CGM training and insertion		1-4 hours
	**Visit 2**	Review of baseline bloods, pump settings and CGM data; adjustment of treatment	2 weeks after Visit 1 (+1 week); Run-in could be repeated	1-2 hours
**Training period**	**Visit 3**	Randomisation, repeat HbA1c if Visit 3 and Visit 1 are >28 days apart, urine pregnancy test, insulin pump refresher training, competency assessment	May coincide with Visit 2, within 8 weeks of Visit 1	3-4 hours
**Usual insulin pump therapy Intervention (6 months)**	**Visit 4***	Initiation of standard therapy arm at clinic/home, glucometer download, recording of current insulin requirements, blinded CGM	4 weeks after Randomisation (±1 week)	2-6 hours
	Contact 1	Study update	Within 24 to 48 hours after Visit 4	<1 hour
	**Visit 5****	Study update	1 week after Visit 4 (±3 days)	<1 hour
	Contact 2	Study update	2 weeks after Visit 4 (±3 days)	<1 hour
	Contact 3	Study update	1 month after Visit 4 (±2 weeks)	<1 hour
	Contact 4	Study update	2 months after Visit 4 (±2 weeks)	<1 hour
	**Visit 6**	3-month visit: HbA1c, urine pregnancy test, glucometer download, recording of current insulin requirements, surveys, blinded CGM	4 months after Randomisation (±2 weeks)	1-3 hours
	Contact 5	Study update	5 months after Randomisation (±2 weeks)	<1 hour
	Contact 6	Study update	6 months after Randomisation (±2 weeks)	<1 hour
	**Visit 7**	Blinded CGM	2-4 weeks before planned Visit 8	<0.5 hour
	**Visit 8**	End of standard pump therapy treatment arm (6 months): HbA1c, glucometer download, recording of current insulin requirements, surveys and focus groups, resume usual care	7 months after Randomisation (±2 weeks)	1-3 hours

* In-person clinic visit mandatory in USA only.
** Could be done via phone/e-mail.
HbA1c, glycated haemoglobin; CGM, continuous glucose monitoring.

For peer review only - http://bmjopen.bmj.com/site/about/guidelines.xhtml

Figure Legends

Figure 1 Study flow chart. HbA1c, glycated haemoglobin; CGM, continuous glucose monitoring.

Figure 2 FlorenceM closed-loop system prototype.

The system consists of a continuous glucose monitoring transmitter with Guardian 3 sensor (Medtronic), an insulin pump (modified 640G pump, Medtronic), and an Android smartphone running the control algorithm (Cambridge).

References

1. International Diabetes Federation. IDF Diabetes Atlas 2017. 8th Edition 2017 http://www.diabetesatlas.org (last accessed 26th July 2018).
2. Wood JR, Miller KM, Maahs DM, Beck RW, DiMeglio LA, Libman IM, et al. Most youth with type 1 diabetes in the T1D Exchange Clinic Registry do not meet American Diabetes Association or International Society for Pediatric and Adolescent Diabetes clinical guidelines. Diabetes Care. 2013;36(7):2035-7.
3. Diabetes Research in Children Network Study G, Buckingham B, Beck RW, Tamborlane WV, Xing D, Kollman C, et al. Continuous glucose monitoring in children with type 1 diabetes. J Pediatr. 2007;151(4):388-93, 93 e1-2.
4. Bode BW, Schwartz S, Stubbs HA, Block JE. Glycemic characteristics in continuously monitored patients with type 1 and type 2 diabetes: normative values. Diabetes Care. 2005;28(10):2361-6.
5. Cryer PE. Diverse causes of hypoglycemia-associated autonomic failure in diabetes. N Engl J Med. 2004;350(22):2272-9.
6. Cryer PE. Mechanisms of hypoglycemia-associated autonomic failure and its component syndromes in diabetes. Diabetes. 2005;54(12):3592-601.
7. Wild D, von Maltzahn R, Brohan E, Christensen T, Clauson P, Gonder-Frederick L. A critical review of the literature on fear of hypoglycemia in diabetes: Implications for diabetes management and patient education. Patient Educ Couns. 2007;68(1):10-5.
8. Kropff J, DeVries JH. Continuous Glucose Monitoring, Future Products, and Update on Worldwide Artificial Pancreas Projects. Diabetes Tech Ther. 2016;18 Suppl 2:S253-63.
9. Juvenile Diabetes Research Foundation Continuous Glucose Monitoring Study G, Tamborlane WV, Beck RW, Bode BW, Buckingham B, Chase HP, et al. Continuous glucose monitoring and intensive treatment of type 1 diabetes. N Engl J Med. 2008;359(14):1464-76.
10. Kordonouri O, Pankowska E, Rami B, Kapellen T, Coutant R, Hartmann R, et al. Sensor-augmented pump therapy from the diagnosis of childhood type 1 diabetes: results of the Paediatric Onset Study (ONSET) after 12 months of treatment. Diabetologia. 2010;53(12):2487-95.
11. Pickup JC, Freeman SC, Sutton AJ. Glycaemic control in type 1 diabetes during real time continuous glucose monitoring compared with self monitoring of blood glucose: meta-analysis of randomised controlled trials using individual patient data. Bmj. 2011;343:d3805.
12. Bergenstal RM, Tamborlane WV, Ahmann A, Buse JB, Dailey G, Davis SN, et al. Effectiveness of sensor-augmented insulin-pump therapy in type 1 diabetes. N Engl J Med. 2010;363(4):311-20.
13. Bergenstal RM, Klonoff DC, Garg SK, Bode BW, Meredith M, Slover RH, et al. Threshold-based insulin-pump interruption for reduction of hypoglycemia. N Engl J Med. 2013;369(3):224-32.
14. Thabit H, Hovorka R. Coming of age: the artificial pancreas for type 1 diabetes. Diabetologia. 2016;59(9):1795-805.
15. Hovorka R. Closed-loop insulin delivery: from bench to clinical practice. Nat Rev Endocrinol. 2011;7(7):385-95.
16. FDA News Release. FDA approves first automated insulin delivery device for type 1 diabetes. http://www.fda.gov/NewsEvents/Newsroom/PressAnnouncements/ucm522974.htm (last accessed 26 July 2018) [
17. Kropff J, Del Favero S, Place J, Toffanin C, Visentin R, Monaro M, et al. 2 month evening and night closed-loop glucose control in patients with type 1 diabetes under free-living conditions: a randomised crossover trial. Lancet Diabetes Endocrinol. 2015;3(12):939-47.
18. Hovorka R, Allen JM, Elleri D, Chassin LJ, Harris J, Xing D, et al. Manual closed-loop insulin delivery in children and adolescents with type 1 diabetes: a phase 2 randomised crossover trial. Lancet. 2010;375(9716):743-51.
19. Elleri D, Allen JM, Kumareswaran K, Leelarathna L, Nodale M, Caldwell K, et al. Closed-loop basal insulin delivery over 36 hours in adolescents with type 1 diabetes: randomized clinical trial. Diabetes Care. 2013;36(4):838-44.

20. Nimri R, Danne T, Kordonouri O, Atlas E, Bratina N, Biester T, et al. The "Glucositter" overnight automated closed loop system for type 1 diabetes: a randomized crossover trial. Pediatr Diabetes. 2013;14(3):159-67.
21. Hovorka R, Elleri D, Thabit H, Allen JM, Leelarathna L, El-Khairi R, et al. Overnight Closed Loop Insulin Delivery in Young People with Type 1 Diabetes: A Free-Living Randomised Clinical Trial. Diabetes Care. 2014;37(5):1204-11.
22. Thabit H, Tauschmann M, Allen JM, Leelarathna L, Hartnell S, Wilinska ME, et al. Home Use of an Artificial Beta Cell in Type 1 Diabetes. N Engl J Med. 2015;373(22):2129-40.
23. Nimri R, Muller I, Atlas E, Miller S, Kordonouri O, Bratina N, et al. Night glucose control with MD-Logic artificial pancreas in home setting: a single blind, randomized crossover trial-interim analysis. Pediatr Diabetes. 2014;15(2):91-9.
24. Tauschmann M, Allen JM, Wilinska ME, Thabit H, Stewart Z, Cheng P, et al. Day-and-Night Hybrid Closed-Loop Insulin Delivery in Adolescents With Type 1 Diabetes: A Free-Living, Randomized Clinical Trial. Diabetes Care. 2016;Jan 6 [Epub ahead of print].
25. Leelarathna L, Dellweg S, Mader JK, Allen JM, Benesch C, Doll W, et al. Day and night home closed-loop insulin delivery in adults with type 1 diabetes: three-center randomized crossover study. Diabetes Care. 2014;37(7):1931-7.
26. Thabit H, Lubina-Solomon A, Stadler M, Leelarathna L, Walkinshaw E, Pernet A, et al. Home use of closed-loop insulin delivery for overnight glucose control in adults with type 1 diabetes: a 4-week, multicentre, randomised crossover study. Lancet Diabetes Endocrinol. 2014;2(9):701-9.
27. Barnard KD, Wysocki T, Allen JM, Elleri D, Thabit H, Leelarathna L, et al. Closing the loop overnight at home setting: psychosocial impact for adolescents with type 1 diabetes and their parents. BMJ Open Diabetes Res Care. 2014;2(1):e000025.
28. Bekiari E, Kitsios K, Thabit H, Tauschmann M, Athanasiadou E, Karagiannis T, et al. Artificial pancreas treatment for outpatients with type 1 diabetes: systematic review and meta-analysis. Bmj. 2018;361:k1310.
29. Wilinska ME, Chassin LJ, Acerini CL, Allen JM, Dunger DB, Hovorka R. Simulation environment to evaluate closed-loop insulin delivery systems in type 1 diabetes. J Diabetes Sci Technol. 2010;4(1):132-44.
30. Boughton CK, Hovorka R. Is an artificial pancreas (closed-loop system) for Type 1 diabetes effective? Diabet Med. 2019;36(3):279-86.
31. Stevens K. Developing a descriptive system for a new preference-based measure of health-related quality of life for children. Qual Life Res. 2009;18(8):1105-13.
32. Wille N, Badia X, Bonsel G, Burstrom K, Cavrini G, Devlin N, et al. Development of the EQ-5D-Y: a child-friendly version of the EQ-5D. Qual Life Res. 2010;19(6):875-86.
33. Effectiveness of continuous glucose monitoring in a clinical care environment: evidence from the Juvenile Diabetes Research Foundation continuous glucose monitoring (JDRF-CGM) trial. Diabetes Care. 2010;33(1):17-22.
34. World Health Organization. Definition, Diagnosis and Classification of Diabetes Mellitus and its Complications. Part 1: Diagnosis and Classification of Diabetes Mellitus. WHO/NCD/NCS/99.2. Geneva, (1999).

For peer review only - http://bmjopen.bmj.com/site/about/guidelines.xhtml

Figure 1. Study flow chart

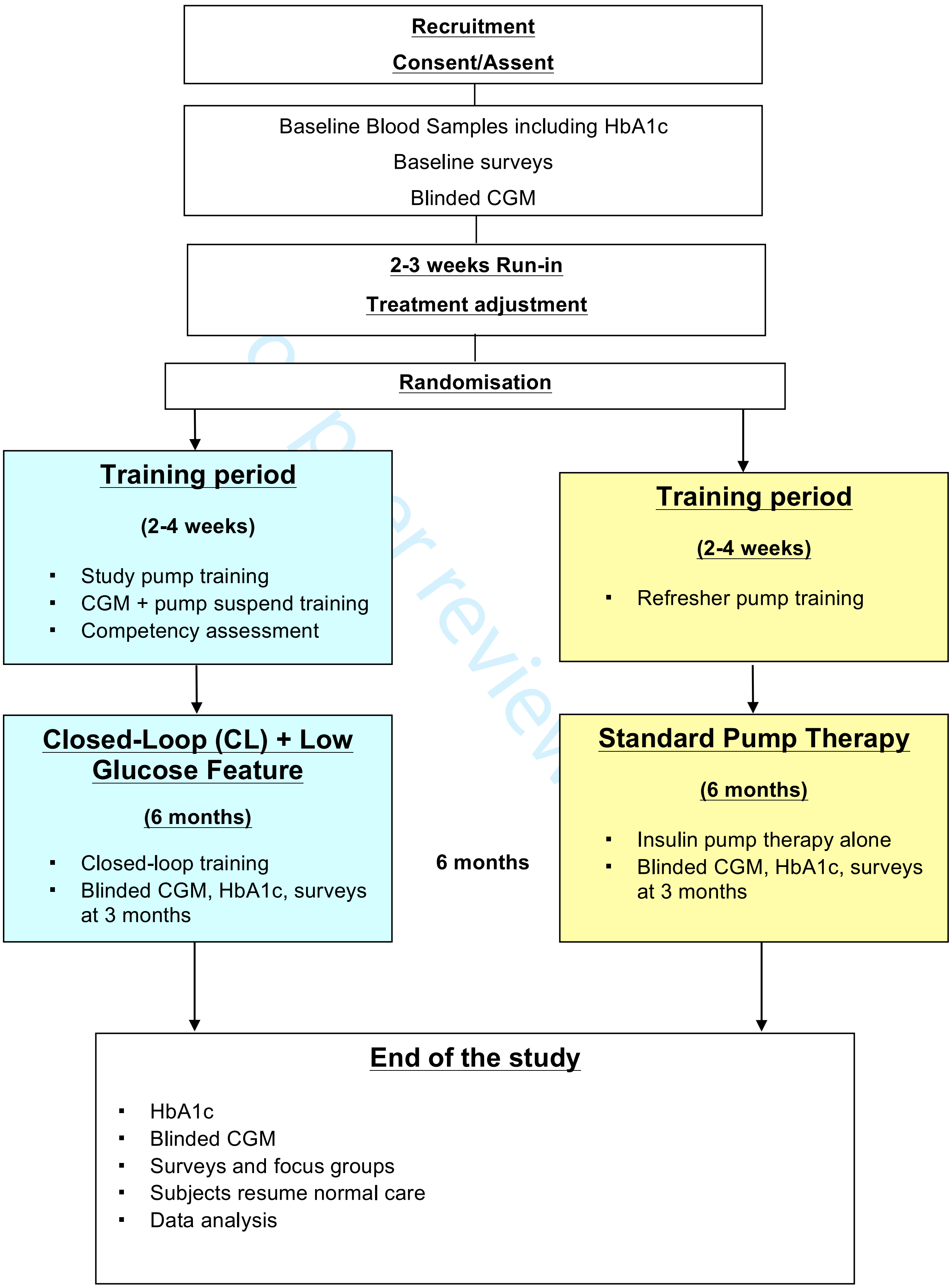

For peer review only - http://bmjopen.bmj.com/site/about/guidelines.xhtml

Figure 2. FlorenceM closed-loop system prototype

The system consists of a continuous glucose monitoring transmitter with Guardian 3 sensor (Medtronic), an insulin pump (modified 640G pump, Medtronic), and an Android smartphone running the control algorithm (Cambridge).

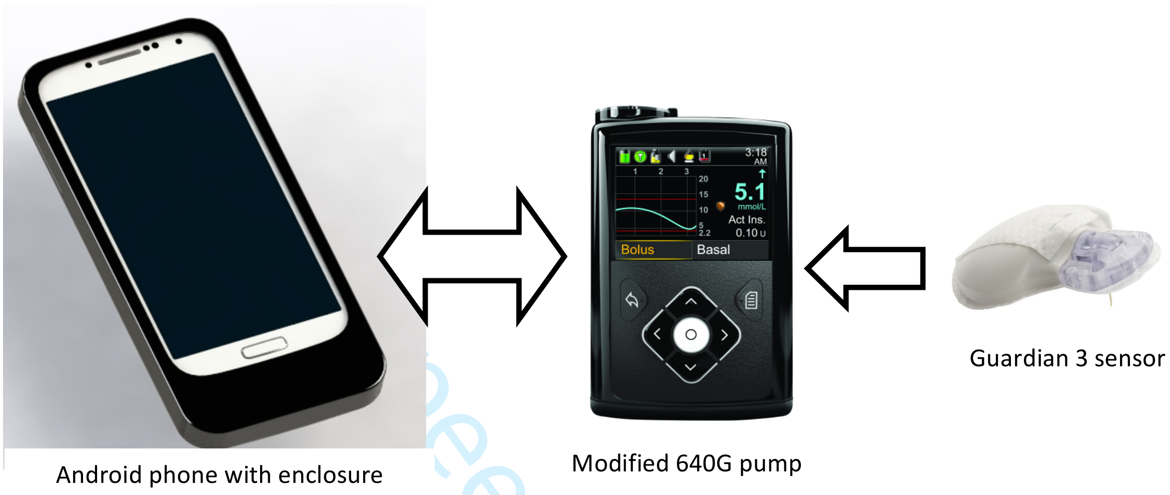

For peer review only

Reporting checklist for protocol of a clinical trial.

Based on the SPIRIT guidelines.

Instructions to authors

Complete this checklist by entering the page numbers from your manuscript where readers will find each of the items listed below.

Your article may not currently address all the items on the checklist. Please modify your text to include the missing information. If you are certain that an item does not apply, please write "n/a" and provide a short explanation.

Upload your completed checklist as an extra file when you submit to a journal.

In your methods section, say that you used the SPIRIT reporting guidelines, and cite them as:

Chan A-W, Tetzlaff JM, Altman DG, Laupacis A, Gøtzsche PC, Krleža-Jerić K, Hróbjartsson A, Mann H, Dickersin K, Berlin J, Doré C, Parulekar W, Summerskill W, Groves T, Schulz K, Sox H, Rockhold FW, Rennie D, Moher D. SPIRIT 2013 Statement: Defining standard protocol items for clinical trials. Ann Intern Med. 2013;158(3):200-207

		Reporting Item	Page Number
Title	#1	Descriptive title identifying the study design, population, interventions, and, if applicable, trial acronym	1
Trial registration	#2a	Trial identifier and registry name. If not yet registered, name of intended registry	2

For peer review only

			NCT02925299 (ClinicalTrials.gov)<br>DAN05
Trial registration: data set	#2b	All items from the World Health Organization Trial Registration Data Set	04/10/2016
Protocol version	#3	Date and version identifier	16.04.2018 (6.0)
Funding	#4	Sources and types of financial, material, and other support	14
Roles and responsibilities: contributorship	#5a	Names, affiliations, and roles of protocol contributors	14
Roles and responsibilities: sponsor contact information	#5b	Name and contact information for the trial sponsor	NCT02925299 (ClinicalTrials.gov)
Roles and responsibilities: sponsor and funder	#5c	Role of study sponsor and funders, if any, in study design; collection, management, analysis, and interpretation of data; writing of the report; and the decision to submit the report for publication, including whether they will have ultimate authority over any of these activities	NCT02925299 (ClinicalTrials.gov)

For peer review only

Roles and responsibilities: committees	#5d	Composition, roles, and responsibilities of the coordinating centre, steering committee, endpoint adjudication committee, data management team, and other individuals or groups overseeing the trial, if applicable (see Item 21a for data monitoring committee)	See supplementary file
Background and rationale	#6a	Description of research question and justification for undertaking the trial, including summary of relevant studies (published and unpublished) examining benefits and harms for each intervention	4
Background and rationale: choice of comparators	#6b	Explanation for choice of comparators	4
Objectives	#7	Specific objectives or hypotheses	2, 5
Trial design	#8	Description of trial design including type of trial (eg, parallel group, crossover, factorial, single group), allocation ratio, and framework (eg, superiority, equivalence, non-inferiority, exploratory)	5
Study setting	#9	Description of study settings (eg, community clinic, academic hospital) and list of countries where data will be collected. Reference to where list of study sites can be obtained	5

For peer review only

Eligibility criteria	#10	Inclusion and exclusion criteria for participants. If applicable, eligibility criteria for study centres and individuals who will perform the interventions (eg, surgeons, psychotherapists)	15
Interventions: description	#11a	Interventions for each group with sufficient detail to allow replication, including how and when they will be administered	5, 16, 17
Interventions: modifications	#11b	Criteria for discontinuing or modifying allocated interventions for a given trial participant (eg, drug dose change in response to harms, participant request, or improving / worsening disease)	See protocol
Interventions: adherence	#11c	Strategies to improve adherence to intervention protocols, and any procedures for monitoring adherence (eg, drug tablet return; laboratory tests)	N/A
Interventions: concomitant care	#11d	Relevant concomitant care and interventions that are permitted or prohibited during the trial	7
Outcomes	#12	Primary, secondary, and other outcomes, including the specific measurement variable (eg, systolic blood pressure), analysis metric (eg, change from baseline, final value, time to event), method of aggregation (eg, median, proportion), and time point for each outcome.	9, 10

For peer review only

		Explanation of the clinical relevance of chosen efficacy and harm outcomes is strongly recommended	
Participant timeline	#13	Time schedule of enrolment, interventions (including any run-ins and washouts), assessments, and visits for participants. A schematic diagram is highly recommended (see Figure)	See Figure 1
Sample size	#14	Estimated number of participants needed to achieve study objectives and how it was determined, including clinical and statistical assumptions supporting any sample size calculations	12
Recruitment	#15	Strategies for achieving adequate participant enrolment to reach target sample size	5
Allocation: sequence generation	#16a	Method of generating the allocation sequence (eg, computer-generated random numbers), and list of any factors for stratification. To reduce predictability of a random sequence, details of any planned restriction (eg, blocking) should be provided in a separate document that is unavailable to those who enrol participants or assign interventions	6

For peer review only

Allocation concealment mechanism	#16b	Mechanism of implementing the allocation sequence (eg, central telephone; sequentially numbered, opaque, sealed envelopes), describing any steps to conceal the sequence until interventions are assigned	6
Allocation: implementation	#16c	Who will generate the allocation sequence, who will enrol participants, and who will assign participants to interventions	
Blinding (masking)	#17a	Who will be blinded after assignment to interventions (eg, trial participants, care providers, outcome assessors, data analysts), and how	N/A
Blinding (masking): emergency unblinding	#17b	If blinded, circumstances under which unblinding is permissible, and procedure for revealing a participant's allocated intervention during the trial	N/A
Data collection plan	#18a	Plans for assessment and collection of outcome, baseline, and other trial data, including any related processes to promote data quality (eg, duplicate measurements, training of assessors) and a description of study instruments (eg, questionnaires, laboratory tests) along with their reliability and validity, if known. Reference to where data	See protocol

For peer review only

		collection forms can be found, if not in the protocol	
Data collection plan: retention	#18b	Plans to promote participant retention and complete follow-up, including list of any outcome data to be collected for participants who discontinue or deviate from intervention protocols	See protocol
Data management	#19	Plans for data entry, coding, security, and storage, including any related processes to promote data quality (eg, double data entry; range checks for data values). Reference to where details of data management procedures can be found, if not in the protocol	12
Statistics: outcomes	#20a	Statistical methods for analysing primary and secondary outcomes. Reference to where other details of the statistical analysis plan can be found, if not in the protocol	9
Statistics: additional analyses	#20b	Methods for any additional analyses (eg, subgroup and adjusted analyses)	11
Statistics: analysis population and missing data	#20c	Definition of analysis population relating to protocol non-adherence (eg, as randomised analysis), and any statistical methods to handle missing data (eg, multiple imputation)	11

For peer review only

Data monitoring: formal committee	#21a	Composition of data monitoring committee (DMC); summary of its role and reporting structure; statement of whether it is independent from the sponsor and competing interests; and reference to where further details about its charter can be found, if not in the protocol. Alternatively, an explanation of why a DMC is not needed	12
Data monitoring: interim analysis	#21b	Description of any interim analyses and stopping guidelines, including who will have access to these interim results and make the final decision to terminate the trial	11
Harms	#22	Plans for collecting, assessing, reporting, and managing solicited and spontaneously reported adverse events and other unintended effects of trial interventions or trial conduct	12-13
Auditing	#23	Frequency and procedures for auditing trial conduct, if any, and whether the process will be independent from investigators and the sponsor	12
Research ethics approval	#24	Plans for seeking research ethics committee / institutional review board (REC / IRB) approval	13
Protocol amendments	#25	Plans for communicating important protocol modifications (eg, changes to eligibility criteria,	13

For peer review only

		outcomes, analyses) to relevant parties (eg, investigators, REC / IRBs, trial participants, trial registries, journals, regulators)	
Consent or assent	#26a	Who will obtain informed consent or assent from potential trial participants or authorised surrogates, and how (see Item 32)	5
Consent or assent: ancillary studies	#26b	Additional consent provisions for collection and use of participant data and biological specimens in ancillary studies, if applicable	N/A
Confidentiality	#27	How personal information about potential and enrolled participants will be collected, shared, and maintained in order to protect confidentiality before, during, and after the trial	12
Declaration of interests	#28	Financial and other competing interests for principal investigators for the overall trial and each study site	14
Data access	#29	Statement of who will have access to the final trial dataset, and disclosure of contractual agreements that limit such access for investigators	12
Ancillary and post trial care	#30	Provisions, if any, for ancillary and post-trial care, and for compensation to those who suffer harm from trial participation	N/A

For peer review only

Dissemination policy: trial results	#31a	Plans for investigators and sponsor to communicate trial results to participants, healthcare professionals, the public, and other relevant groups (eg, via publication, reporting in results databases, or other data sharing arrangements), including any publication restrictions	9, 13
Dissemination policy: authorship	#31b	Authorship eligibility guidelines and any intended use of professional writers	See supplementary file
Dissemination policy: reproducible research	#31c	Plans, if any, for granting public access to the full protocol, participant-level dataset, and statistical code	
Informed consent materials	#32	Model consent form and other related documentation given to participants and authorised surrogates	Approved consents for UK and USA available
Biological specimens	#33	Plans for collection, laboratory evaluation, and storage of biological specimens for genetic or molecular analysis in the current trial and for future use in ancillary studies, if applicable	8

The SPIRIT checklist is distributed under the terms of the Creative Commons Attribution License CC-BY-ND 3.0. This checklist can be completed online using https://www.goodreports.org/, a tool made by the EQUATOR Network in collaboration with Penelope.ai

For peer review only

BMJ Open

Assessing the efficacy, safety and utility of 6 month day-and-night automated closed-loop insulin delivery under free living conditions compared to insulin pump therapy in children and adolescents with type 1 diabetes: an open-label, multi-centre, multi-national, single-period, randomised, parallel group study protocol

Journal:	*BMJ Open*
Manuscript ID	bmjopen-2018-027856.R2
Article Type:	Protocol
Date Submitted by the Author:	20-Apr-2019

Complete List of Authors:	Musolino, Gianluca; Wellcome Trust-MRC Institute of Metabolic Science, University of Cambridge; University of Cambridge, Department of Paediatrics<br>Allen, Janet; Wellcome Trust-MRC Institute of Metabolic Science, University of Cambridge ; University of Cambridge, Department of Paediatrics<br>Hartnell, Sara; Cambridge University Hospitals NHS Foundation Trust, Department of Diabetes and Endocrinology<br>Wilinska, Malgorzata; Wellcome Trust-MRC Institute of Metabolic Science, University of Cambridge ; University of Cambridge, Department of Paediatrics<br>Tauschmann, Martin; Wellcome Trust-MRC Institute of Metabolic Science, University of Cambridge; Medical University of Vienna, Department of Pediatrics and Adolescent Medicine<br>Boughton, Charlotte; Wellcome Trust-MRC Institute of Metabolic Science, University of Cambridge<br>Campbell, Fiona; Leeds Children's Hospital, Department of Paediatric Diabetes<br>Denvir, Louise; Nottingham University Hospitals NHS Trust, Department of Paediatric Diabetes and Endocrinology<br>Trevelyan, Nicola; Southampton Children's Hospital, Southampton General Hospital<br>Wadwa, Paul; Barbara Davis Center for Childhood Diabetes, University of Colorado<br>DiMeglio, Linda; Indiana University School of Medicine, Division of Pediatric Endocrinology and Diabetology and Wells Center for Pediatric Research, Department of Pediatrics<br>Buckingham, Bruce A.; Stanford University, Division of Pediatric Endocrinology<br>Weinzimer, Stuart; Yale University, Department of Pediatrics<br>Acerini, Carlo; Wellcome Trust-MRC Institute of Metabolic Science, University of Cambridge ; University of Cambridge, Department of Paediatrics<br>Hood, Korey; Stanford University, Division of Pediatric Endocrinology<br>Fox, Steven; University of Southern California, Department of

	Pharmaceutical and Health Economics, School of Pharmacy<br>Kollman, Craig; Jaeb Center for Health Research<br>Sibayan, Judy; Jaeb Center for Health Research<br>Borgman, Sarah; Jaeb Center for Health Research<br>Cheng, Peiyao; Jaeb Center for Health Research<br>Hovorka, Roman; Wellcome Trust-MRC Institute of Metabolic Science, University of Cambridge ; University of Cambridge, Department of Paediatrics
**Primary Subject Heading**:	Paediatrics
Secondary Subject Heading:	Diabetes and endocrinology
Keywords:	Type 1 diabetes, Closed-loop, Artificial pancreas

SCHOLARONE™ Manuscripts

Assessing the efficacy, safety and utility of 6 month day-and-night automated closed-loop insulin delivery under free living conditions compared to insulin pump therapy in children and adolescents with type 1 diabetes: an open-label, multi-centre, multi-national, single-period, randomised, parallel group study protocol

[1,3]Musolino G, [1,3]Allen JM, [2]Hartnell S, [1,3]Wilinska ME, [1,4]Tauschmann M, [1]Boughton C, [5]Campbell F, [6]Denvir L, [7]Trevelyan N, [8]Wadwa RP, [9]DiMeglio LA, [10]Buckingham BA, [11]Weinzimer SA, [1,3]Acerini CL, [10]Hood KK, [12]Fox DS, [13]Kollman C, [13]Sibayan J, [13]Borgman S, [13]Cheng P, and [1,3]Hovorka R on behalf of DAN05 Consortium

[1]Wellcome Trust-MRC Institute of Metabolic Science, University of Cambridge, Cambridge, UK

[2]Department of Diabetes & Endocrinology, Cambridge University Hospitals NHS Foundation Trust, Cambridge, UK

[3]Department of Paediatrics, University of Cambridge, Cambridge, UK

[4]Department of Pediatrics and Adolescent Medicine, Medical University of Vienna, Austria

[5]Leeds Children's Hospital, Department of Paediatric Diabetes, Leeds, UK

[6]Department of Paediatric Diabetes and Endocrinology, Nottingham University Hospitals NHS Trust, Nottingham, UK

[7]Southampton Children's Hospital, Southampton General Hospital, Southampton, UK

[8]Barbara Davis Center for Childhood Diabetes, University of Colorado, Aurora, CO, USA

[9]Division of Pediatric Endocrinology and Diabetology and Wells Center for Pediatric Research, Department of Pediatrics, Indiana University School of Medicine, Indianapolis, IN, USA

[10]Division of Pediatric Endocrinology, Stanford University, Stanford, CA, USA

[11]Department of Pediatrics, Yale University, New Haven, CT, USA

[12]Department of Pharmaceutical and Health Economics, School of Pharmacy, University of Southern California, Los Angeles, CA, USA

[13]Jaeb Center for Health Research, Tampa, FL, USA

Correspondence

Roman Hovorka, PhD

University of Cambridge Metabolic Research Laboratories and NIHR Cambridge Biomedical Research Centre,

Wellcome Trust-MRC Institute of Metabolic Science, Box 289,

Addenbrooke's Hospital, Hills Road, Cambridge CB2 0QQ, UK

Tel: +44 1223 762 862, fax: +44 1223 330 598, e-mail: rh347@cam.ac.uk

Key Words: Type 1 diabetes, Closed-loop, Artificial pancreas

Word count abstract: 299 (maximum 300)

Word count paper: 4000 (maximum 4000)

ABSTRACT

Introduction

Closed-loop systems titrate insulin based on sensor glucose levels, providing novel means to reduce the risk of hypoglycaemia while improving glycaemic control. We will assess effectiveness of 6-month day-and-night closed-loop insulin delivery compared to usual care (conventional or sensor-augmented pump therapy) in children and adolescents with type 1 diabetes.

Methods and analysis

The trial adopts an open-label, multi-centre, multi-national (UK and USA), randomised, single-period, parallel design. Participants (n=130) are children and adolescents (age ≥ 6 and <19 years) with type 1 diabetes for at least 1 year, and insulin pump use for at least 3 months with sub-optimal glycaemic control [glycated haemoglobin ≥58mmol/mol (7.5%) and ≤86mmol/mol (10%)]. After a 2-3 week run-in period, participants will be randomised to 6-month use of hybrid closed-loop insulin delivery, or to usual care. Analyses will be conducted on an intention to treat basis. The primary outcome is glycated haemoglobin at 6 months. Other key endpoints include time in the target glucose range (3.9 to 10mmol/l, 70 to 180mg/dl), mean sensor glucose, and time spent above and below target. Secondary outcomes include standard deviation and coefficient of variation of sensor glucose levels, time with sensor glucose levels <3.5mmol/l (63mg/dl) and <3.0mmol/l (54mg/dl), area under the curve of glucose <3.5mmol/l (63mg/dl), time with glucose levels >16.7mmol/l (300mg/dl), area under the curve of glucose >10.0mmol/l (180mg/dl), total, basal and bolus insulin dose, body mass index z-score, and blood pressure. Cognitive, emotional and behavioural characteristics of participants and caregivers and their responses to the closed-loop and clinical trial will be assessed. An incremental cost-effectiveness ratio (ICER) for closed-loop will be estimated.

Ethics and dissemination

Cambridge South Research Ethics Committee and Jaeb Center for Health Research Institutional Review Office approved the study. The findings will be disseminated by peer-review publications and conference presentations.

Trial registration NCT02925299 (ClinicalTrials.gov).

For peer review only - http://bmjopen.bmj.com/site/about/guidelines.xhtml

Strengths and limitations of this study

- The study adopts an open-label, multi-centre, multi-national, randomised, parallel design: it includes a large group of children and adolescents across wide geographical locations
- The trial adopts a 6-month follow-up period of hybrid closed-loop insulin delivery during unrestricted living
- Participants in the two study groups will have an equal number of study visits
- The study design excludes participants with recurrent incidents of severe hypoglycaemia or diabetic ketoacidosis during the previous 6 months, living alone, and those with glycated haemoglobin below 58mmol/mol (7.5%) and above 86mmol/mol (10%) and with high or very low daily insulin requirements (total daily insulin dose ≥2IU/kg/day or <15IU/day)
- All participants are already pump users, somewhat limiting generalizability

For peer review only - http://bmjopen.bmj.com/site/about/guidelines.xhtml

INTRODUCTION

Type 1 diabetes is characterised by a deficiency of insulin caused by immunologically-mediated damage to pancreatic beta cells, leading to raised blood glucose levels. Diabetes is one of the most common metabolic conditions. It is estimated that in 2017 1,100,000 children and adolescents (0-19 years) worldwide had type 1 diabetes and that the number of newly diagnosed cases was over 130,000 (1). The incidence rate in children is increasing by approximately 3-4% per year with geographic differences (1). Earlier onset can result in diabetes complications appearing at a younger age, whilst dependence on lifelong insulin imposes a heavy burden on children, carers as well as health care systems.

Despite continuing progress, glycaemic control in children and adolescents with type 1 diabetes remains suboptimal (2). The achievement of recommended treatment goals is limited by the risk of hypoglycaemia. Even in those with the desired level of glycaemic control, non-physiological glucose excursions occur with periods of silent hyper- and hypoglycaemia (3, 4). Individuals have blunted counter-regulatory responses to hypoglycaemia impairing recovery and increasing the threat of future episodes (5). Recurrent episodes may lead to hypoglycaemic unawareness, increasing the risk of severe hypoglycaemia (6). Hypoglycaemia has psychological consequences including the fear of hypoglycaemia with resulting maladaptive coping behaviours, such as excessive eating or under-insulinising, that may negatively impact glycaemic control (7).

The development of continuous glucose monitoring has been a major advance (8-11). Sensor-augmented pumps combine real-time continuous glucose monitoring with insulin pump (12). Insulin pumps with low glucose suspend feature have been shown to reduce hypoglycaemia (13). These systems, however, overall provide little or no automation to adjust insulin delivery to match glucose excursions.

An artificial pancreas (a closed-loop system) adjusts insulin automatically and represents a realistic treatment option for type 1 diabetes (14). The closed-loop control algorithm translates, in real-time, sensor glucose levels received from the glucose monitoring device and computes the amount of insulin to be delivered by the coupled insulin pump. Hybrid closed-loop systems automatically titrate insulin delivery although the user manages insulin boosts at meal time (15). In 2017, the first closed-loop system entered clinical use in the USA (16).

Closed-loop systems may improve glycaemic control while reducing the risk of hypoglycaemia (17). They have been evaluated in children and adolescents under controlled laboratory conditions (18-20) and in home settings (21-24). Investigations in adults have also been conducted (22, 25, 26). Psychosocial assessments support acceptability and benefits of this therapeutic approach among children/adolescents and carers (27). Closed-loop systems are associated with increased time in near normoglycaemia and reduced time in hypoglycaemia and hyperglycaemia (28). So far, evaluations have been limited to 3 months (22).

The present study will assess the efficacy, safety, utility and acceptability of 6-month day-and-night hybrid closed-loop insulin delivery during unrestricted living in comparison to usual care in children and adolescents with type 1 diabetes.

For peer review only - http://bmjopen.bmj.com/site/about/guidelines.xhtml

METHODS AND ANALYSIS

Overview

This trial adopts an open-label, multi-centre, multi-national, single-period, randomised, parallel group design, involving a 6-month home study period during which day-and-night glucose levels will be managed either by a closed-loop system (intervention group) or by insulin pump therapy (control group) (Figure 1). We aim to recruit up to 150 children and adolescents aged ≥6 to <19 years with type 1 diabetes on insulin pump therapy (approximately equal proportion of those aged ≥6 to 12 years and 13 to <19 years, a minimum quota of 25% participants with baseline glycated haemoglobin >69mmol/mol, >8.5%). Inclusion and exclusion criteria are summarised in Table 1.

The University of Cambridge (UK) and Jaeb Center for Health Research (USA) are the coordinating centres. Clinical centres include:

1) Addenbrooke's Hospital, Cambridge, UK
2) Barbara Davis Center for Childhood Diabetes, Aurora, USA
3) Indiana University, Indianapolis, USA
4) Leeds Teaching Hospital, Leeds, UK
5) Nottingham Children's Hospital, Nottingham, UK
6) Southampton Children's Hospital, Southampton, UK
7) Stanford University, Stanford, California, USA
8) Yale University, New Haven, Connecticut, USA

Cognitive, emotional, and behavioural characteristics of participants and family members and their response to the closed-loop will be assessed gathering both quantitative (validated surveys) and qualitative data (focus groups). Written informed consent/assent will be obtained from all participants and guardians before any study-related activities.

Study schedule

The study will comprise up to 8 visits and 6 telephone/email contacts (see Table 2 and Table 3). The maximum study duration is 8 months.

Screening and baseline assessment

At screening, blood samples for full blood count, liver, thyroid function and anti-transglutaminase antibodies (with IgA levels if not done within previous 12 months) will be taken. Non-hypoglycaemia C-peptide, glucose and glycated haemoglobin will be measured and a urine pregnancy test in females of child-bearing potential will be performed. Surveys investigating participants' quality of life, psychosocial and cognitive functioning, and response to their current treatment will be distributed. Participants will be fitted with a blinded continuous glucose monitoring device (Libre Pro, Abbott Diabetes Care, Alameda, CA, USA) that will be worn during the run-in period at home for up to 14 days.

Run-in period

During a 2-3 week run-in period, subjects will continue using their own insulin pump. Data obtained from blinded glucose sensors and pump downloads may be utilised for treatment adjustments. The run-in period may be extended/repeated if no or limited sensor data is

For peer review only - http://bmjopen.bmj.com/site/about/guidelines.xhtml

available. At least 10 days of sensor data need to be collected. A longer run-in will not be used for additional fine-tuning of treatment adjustments.

Randomisation

Central randomisation software will be used with stratification by site and baseline glycated haemoglobin. The randomisation ratio will be 1:1 within each stratum. The randomisation list created by the study statistician is encrypted.

Treatment period

1. Automated day-and-night hybrid closed-loop insulin delivery combined with low glucose suspend feature (interventional arm)

Participants allocated to the closed-loop group will be trained on using the study insulin pump (modified Medtronic 640G pump, Medtronic, Northridge, CA, USA) and real-time continuous glucose sensor (Guardian 3, Medtronic). This represents a complex intervention over usual care, especially for subjects under pump therapy alone. Once deemed competent with the use of the devices, participants will receive training required for the closed-loop system. Competency on the use of closed-loop will be evaluated. During closed-loop period, participants will program meal boluses estimating ingested carbohydrate amounts. Specific instructions during closed-loop related to exercise management, sick day rules, hypo- and hyperglycaemia management and technical troubleshooting will be provided.

2. Usual care (conventional or sensor-augmented pump therapy) (control arm)

Participants in control arm will receive refresher training on key aspects of insulin pump therapy (advanced boluses, temporary basal, infusion set change, sensor calibrations). During 6-month control intervention period, subjects will continue using either their own insulin pump alone or combined with their pre-study glucose monitoring device.

At the study initiation visit, participants in both study groups will be fitted with a blinded continuous glucose monitoring system (Libre Pro) that will be worn for up to 14 days. If the sensor fails or gets detached, another sensor may be inserted. The sensor data may be used to optimise insulin delivery.

Assessments at 3 months and 6 months

A blood sample will be collected for measurement of glycated haemoglobin. A urine pregnancy test in females of child-bearing potential will be performed. As per usual clinical practice, glucometer downloads and pump data will be reviewed, and adjustments to insulin pump settings will be made as required. Validated surveys evaluating the impact of the devices employed on quality of life, psychosocial and cognitive functioning, diabetes management and treatment satisfaction will be administered. At the 3-month follow-up visit, participants in both study groups will be fitted with blinded continuous glucose monitoring systems (Libre Pro). For assessment of glycaemic control during the final 3-month period of the trial, participants in both study groups will be fitted with a blinded continuous glucose monitoring system 2 to 4 weeks before the end of study. At the 6-month visit, the same procedures as at the 3-month visit will be followed. A subset of subjects/guardians will be invited to join follow-up focus groups.

For peer review only - http://bmjopen.bmj.com/site/about/guidelines.xhtml

Study contacts during 6-month study period

Participants in the two study groups will have an equal number of contact visits. The first planned contact will occur within 24-48 hours after study initiation visit. During the first 2 weeks of the study period, participants will be contacted weekly. Thereafter, participants will be contacted monthly. Subjects/parents and/or the clinical team are free to adjust insulin therapy, but no active treatment optimisation will be undertaken by the research team.

Devices download

As per usual care, insulin pump and blood glucose meter will be downloaded (Medtronic CareLink) every clinic visit (at least every 3 months).

Closed-loop system

The FlorenceM closed-loop system (Figure 2) incorporates a computer-based algorithm hosted by an Android smartphone, which interacts wirelessly with the modified investigational-use-only 640G pump through a proprietary translator device included in the smartphone's enclosure. By using the information received from the glucose sensor, every ten minutes the system computes a new temporary basal insulin infusion rate, which is automatically sent to the insulin pump. The treat-to-target control algorithm aims to achieve a default glucose level of 5.8mmol/l (104mg/dl) and regulates the actual level depending on fasting versus postprandial status and the accuracy of model-based glucose predictions. No remote monitoring is planned. While the system is charging and connected to internet, the device uploads data on a server. The study pump comprises continuous glucose monitoring receiver and provides hypoglycaemia and hyperglycaemia alarms, which can be activated/ personalised by the participants.

Safety precautions during closed-loop

Participants will be asked to perform capillary calibrations before breakfast and dinner. If sensor glucose value is >3.0mmol/l (54mg/dl) different from capillary glucose level, the sensor will be recalibrated. These directions are based on an in-silico simulation of hypo- and hyperglycaemia risk using the validated Cambridge simulator (29). If sensor glucose becomes unavailable or the smartphone is not in range/operational, the pump will automatically deliver the pre-programmed insulin as set on the pump within 30 minutes. Safety rules limit maximum insulin infusion and suspend insulin delivery when sensor glucose is ≤4.3mmol/l (77mg/dl) or when glucose is rapidly decreasing. In case of a communication failure between control algorithm device and the study pump, the low-glucose feature will interrupt insulin delivery, provided sensor glucose is available. Low glucose suspend/predictive low glucose management will be initially set to suspend insulin delivery at sensor glucose values of 3.9mmol/l (70mg/dl) or less, after which the setting could range from 2.8 to 5.0mmol/l (50mg/dl to 90mg/dl). Predictive low glucose suspend will not be used. Insulin delivery will be resumed in accordance of the low glucose suspend feature implemented on the study pump. A 24-hour local telephone helpline will be available for any technical device issues or problems related to diabetes management.

Participant withdrawal criteria

The following pre-randomisation withdrawal criteria will apply:

For peer review only - http://bmjopen.bmj.com/site/about/guidelines.xhtml

1. Subject/caregiver is unable to demonstrate safe use of study insulin pump as judged by the investigator
2. Subject/caregiver fails to demonstrate compliance with insulin pump and capillary self-monitoring of blood glucose during run-in

Pre- and post-randomisation withdrawal criteria will comprise:

3. Subjects/caregivers may terminate participation in the study at any time without necessarily giving a reason and without any personal disadvantage
4. Significant protocol violation or non-compliance
5. Two distinct episodes of severe hypoglycaemia
6. Two distinct episodes of diabetic ketoacidosis unrelated to infusion site failure and related to the use of the closed-loop
7. Decision by the investigator or the sponsor that termination is in the subject's best medical interest
8. Allergic reaction to insulin
9. Allergic reaction to adhesive surface of infusion set or glucose sensor
10. Subject becomes pregnant during the study period

Subjects withdrawn due to reasons 4-10 will be invited to provide blood sample at the end of the planned study intervention for the assessment of glycated haemoglobin.

Psychosocial evaluations

Cognitive, emotional, and behavioural characteristics of participating subjects and family members and their response to the closed-loop system and clinical trial will be assessed using validated surveys and focus groups. Surveys will be completed at baseline (prior to randomisation), at 3, and 6 months.

To assess how strongly participants value the benefits of the closed-loop (compared with the usual care), we will conduct a discrete choice experiment (DCE). In the DCE, respondents will answer a series of binary choice questions (e.g., "Given a choice between option A or B, which would you prefer...") where those two options offer differing strengths and weaknesses. By varying the performance levels of these different desirable characteristics, we can assess their relative importance.

Focus groups will be completed at the end of the study (6 months). We will conduct virtual focus groups using HIPAA-approved software supported by Stanford University. Focus groups will be run with 3-6 participants and we will work from a script of open-ended questions used to gather feedback and reactions to the closed-loop system/insulin pump therapy, the clinical trial and quality of life changes. Sessions will be audio- and video-taped and transcribed by a professional transcription service.

Blood samples

Screening blood samples will be measured locally. Additional blood samples will be taken for the measurement of non-hypoglycaemia C-peptide and glycated haemoglobin at a central laboratory. Glycated haemoglobin will be assessed at baseline, 3, and 6 months. At each time point, glycated haemoglobin will be measured locally (clinical care) and centrally (analysis of study endpoints). The central analysis will be performed using an International Federation of Clinical Chemistry and Laboratory Medicine aligned method.

For peer review only - http://bmjopen.bmj.com/site/about/guidelines.xhtml

Patient and Public Involvement

The research question and study endpoints are based on feedback from participants of previous studies and in line with prioritising by stakeholders (30). The study design and the assessment of the burden of the intervention were reviewed by focus groups. Results will be disseminated to participants and general public through social media and will be made available on the sponsor's website.

Statistical analysis

Primary Outcome Analysis

The primary analysis will follow the intention-to-treat principle. Data from all randomised subjects will be analysed in the group to which the subjects were assigned through randomisation regardless of the actual treatment received. Data will not be truncated due to protocol deviations.

The primary analysis will evaluate between group differences in glycated haemoglobin levels at the end of treatment period. A 5% significance level will be considered statistically significant for the primary outcome comparison.
Means ± standard deviation (SD) values or percentiles appropriate to the distribution will be reported for the primary outcome by treatment group. The two treatment groups will be compared using a linear regression model adjusting for glycated haemoglobin at baseline, age, and clinical centre as random effect. A 95% confidence interval will be reported for the difference between the randomisation groups based on the linear regression model. Residual values will be examined for an approximate normal distribution. If values are highly skewed, then a transformation or robust statistical methods (e.g., non-parametric or MM estimation) will be used instead. A detailed analysis plan will be provided separately.

Other Key Endpoints

For the following key endpoints at 6 months, the familywise type I error rate will be controlled at two-sided $\alpha = 0.05$. A gatekeeping strategy will be used, where the primary endpoint will be tested first, if passing the significance testing, other key endpoints will be tested in the order listed below using the fixed-sequence method at $\alpha = 0.05$.

- Time spent in the target glucose range from 3.9 to 10.0mmol/l (70 to 180mg/dl)
- Mean sensor glucose
- Time spent above target glucose 10.0mmol/l (180mg/dl)
- Time spent below target glucose 3.9mmol/l (70mg/dl)

If a non-significant ($p>0.05$) result is obtained for any outcome on this list, no further hypothesis testing will be performed for any metrics further down on the list.

Secondary Efficacy Analyses

For these exploratory analyses, the false discovery rate will be used to account for multiple comparisons:
<u>Continuous glucose monitoring derived indices</u>

- Standard deviation of sensor glucose
- Sensor glucose variability measured with the coefficient of variation
- The time with glucose <3.5mmol/l (63mg/dl)

- The time with glucose <3.0mmol/l (54mg/dl)
- Area under the curve of glucose below 3.5mmol/l (63mg/dl)
- The time spent in significant hyperglycaemia (glucose >16.7mmol/l, 300mg/dl)
- Area under the curve of glucose above 10.0mmol/l (180mg/dl)

The following sensor glucose metrics will also be calculated separately for day-time period (06:00-23:59) and night-time period (00:00-05:59):

- The time with glucose from 3.9 to 10.0mmol/l (70-180mg/dl)
- Mean glucose
- Glucose variability as measured by standard deviation
- The time with glucose <3.5mmol/l (63mg/dl)

Binary metrics for glycated haemoglobin

- HbA1c <53mmol/mol (7.0%)
- HbA1c <58mmol/mol (7.5%)
- Relative reduction ≥10% from baseline
- Absolute reduction ≥0.5% from baseline
- Absolute reduction ≥1% from baseline
- Absolute reduction ≥1% from baseline or HbA1c <53mmol/mol (7.0%)

Insulin and other endpoints

- Total, basal and bolus insulin dose
- Body weight (BMI z-score)
- Blood pressure

The above described glycaemic metrics will be based on sensor glucose levels collected during post-randomisation periods of blinded sensors wear.

Safety analyses

The following events will be recorded and compared between treatment groups:

- Number of severe hypoglycaemia events per subject and incidence rate per 100 person-years
- Number of diabetic ketoacidosis events per subject and incidence rate per 100 person-years
- Sensor glucose-measured hypoglycaemic events per week (>15 minutes with glucose <3mmol/l, 54mg/dl)
- Sensor glucose-measured hyperglycaemic events per week (>15 minutes with glucose >16.7mmol/l, 300mg/dl)
- Proportion of subjects with worsening of glycated haemoglobin from baseline to 6 months by >0.5%

If we record enough observed events to allow formal statistical modelling for above safety outcomes, we will perform the following analyses. Poisson regression models will be constructed to compare the treatment group difference for event rates by adjusting for age, baseline glycated haemoglobin and random site effect. If any outlier exists, a robust Poisson regression model will be used instead. For binary glycated haemoglobin outcome, logistic

regression models will be used to compare the treatment group difference by adjusting for age, baseline glycated haemoglobin and random site effect.

Utility assessments

The following system use/function outcomes in the intervention arm will be tabulated:

- Number of low glucose suspend events
- Percentage of time when closed-loop system use is functioning
- Percentage of time when continuous glucose monitoring is used

Subgroup analyses

No subgroups were considered during the power calculations. Interpretation of any subgroup analyses will depend on whether the overall analysis demonstrates a significant treatment group difference. In the absence of such difference, if performed, the subgroup analyses will be interpreted with caution.

Psychosocial analyses

Quantitative data on usability and satisfaction will be analysed using simple descriptive statistics. Additionally, we will analyse scores from the cognitive, emotional, and behavioural assessments to determine if changes occur over time and between groups.

We will construct predictive models in the general linear framework to examine the associations with primary outcomes. For the discrete choice experiment (DCE), the strength of preference (importance) of each performance attribute will be estimated from the pooled DCE responses using standard regression analysis techniques.

Qualitative data will be analysed using Atlas.ti (release 6.0; Scientific Software Development GmbH, Berlin, Germany) to organize and manage the entire corpus of focus group data.

Cost utility analyses

To inform reimbursement and other policy decision-making, we will conduct a cost utility analysis on the benefits of closed-loop. The analysis timeframe for both costs and benefits will include not just the study period, but also anticipated future impacts. Costs will be denominated in US Dollars. They will be framed to include both health-related expenditures and any realised or projected incremental health cost savings. Utility will be quantified in quality adjusted life years (QALYS). We will elicit health related quality of life (HRQOL) during the study period using two preference based measures of health status: the Child Health Utility 9D (31) and the EuroQol 5D-Y (32). Future health and cost impacts, beyond the study period, will be estimated using numerical modelling. Incremental cost effectiveness ratios, comparing the closed-loop system to usual care will be calculated.

Interim analysis

We will not perform an interim analysis.

For peer review only - http://bmjopen.bmj.com/site/about/guidelines.xhtml

Per-protocol analysis

We will conduct a per-protocol analysis in order to replicate the primary analysis, but limited to participants who did not withdraw from the study (withdrawals excluded even if they return for a 6-month glycated haemoglobin measurement) and used closed-loop for at least 70% of the time (intervention group).

Power calculation

Data from the JDRF Continuous Glucose Monitoring Randomised Clinical Trial (33) from subjects who would have met the eligibility criteria for the current trial were used to project the distribution of baseline and 6-month glycated haemoglobin. Among N=53 subjects meeting the eligibility criteria in the JDRF CGM RCT (n=20 subjects 8 to 12 years of age and n=33 subjects 13 to 18 years of age), the upper limit of the confidence interval for the effective SD of glycated haemoglobin was 0.71%. With this effective SD, for a true 0.4% reduction in glycated haemoglobin, power = 85%, 2-sided type 1 error = 5%, 1:1 randomisation, total sample size is estimated to be 116. Adding 10% for potential dropout/non-compliance results in a final total sample size of approximately 128 (64 in each treatment group).

STUDY MANAGEMENT

Data Safety Monitoring Board

A Data Safety Monitoring Board (DSMB) will be instituted. The DSMB will be notified of all serious adverse events and any unanticipated adverse device effects/events and will perform regular safety data review. The DSMB will report to the National Institute of Diabetes and Digestive and Kidney Diseases (the Funder) any safety concerns and recommendations for suspension or early termination of the trial.

Study sponsors

In the UK the study sponsors are the University of Cambridge and the Cambridge University Hospitals NHS Foundation Trust. Study sponsor in the USA is the Jaeb Center for Health Research.

Study management committee

A study management committee composed of the Chief Investigator, Study Coordinators, and Study Data Manager will meet monthly to discuss the operational aspects of the trial.

Data management and monitoring

Designated personnel from Coordinating Centres will be responsible for maintaining quality assurance and quality control systems to ensure that the trial is conducted and data are generated, documented and reported in compliance with the protocol, Good Clinical Practice, and regulatory requirements.

We will observe confidentiality of subject data. Personal details for each participant with a link to a unique identification number will be held locally on a study screening log in the Trial Master File at each of the investigation centres. These details will not be disclosed at any other stage during the study, and all individual results will remain anonymous.

Indemnity

Indemnity for any harm rising on the conduct of research will be provided according to arrangements in respective countries:

1) UK - any liability arising from study design will be covered by clinical trial insurance policy organised by the University of Cambridge. National Health Service indemnity cover will apply for any claims arising from management and conduct of research.

2) USA - any liability arising from study design will be under the responsibility of the participants or their insurance company.

ETHICS AND DISSEMINATION

Approval from independent Research Ethics Committee/Institutional Review Board (UK, East of England-Cambridge South Research Ethics Committee, #16/EE/0380; USA, Jaeb Center for Health Research Institutional Review Board certified by the Office for Human Research Protections, FWA #00000024) has been obtained. The study has undergone a review by regulatory authorities in the UK (Medicines and Healthcare products Regulatory Agency) and in the USA (Food and Drug Administration). All participants will be provided with oral and written information about the trial and procedures involved in the study before obtaining written informed consent. For minors, parents/guardians will provide written informed consent, and written assent will be gained.

Standard operating procedures for monitoring and reporting of all adverse events and adverse device effects will be in place including serious adverse events, serious adverse device effects and specific adverse events, such as severe hypoglycaemia and significant hyperglycaemia with ketosis.

Any substantial amendments to the protocol and other documents shall be submitted to, and approved by, the independent Research Ethics Committee/Institutional Review Board and the regulatory authorities, prior to implementation as per nationally agreed guidelines.

The study started enrolling participants in June 2017 and is expected to complete clinical follow up by November 2019 and to report results in 2020. Trial results will be disseminated in internationally peer-reviewed scientific journals.

For peer review only - http://bmjopen.bmj.com/site/about/guidelines.xhtml

Acknowledgements

Jasdip Mangat supported development and validation of closed-loop system. Josephine Hayes, Matthew Haydock and Nicole Ashcroft (Institute of Metabolic Science, University of Cambridge) provided administrative support. NIHR Cambridge Clinical Research Facility will support the research team in their research-related activities. Artificial Pancreas focus group contributors provided feedback on the study design. West Midlands Young Persons Advisory Group reviewed Participant Information Sheets.

Contributors

RH, MEW, FC, LD, NT, RPW, LADM, BAB, SAW, CLA, and KKH co-designed the study. CK and PC designed the statistical plan. GM, JMA, SH, MT, CB, FC, LD, NT, RPW, LADM, BAB, SAW, and CLA screened and enrolled participants, arranged informed consent from the participants, provided patient care, and took samples. KKH devised the human factors assessments. JS, SB coordinated the study. JS managed randomisation. DSF will conduct the cost utility analysis. RH designed and implemented the glucose controller. GM and RH wrote the manuscript. All authors critically reviewed the report. No writing assistance was provided.

Funding

National Institute of Diabetes and Digestive and Kidney Diseases of the National Institutes of Health under Award Number UC4DK108520. Additional support for the artificial pancreas work is from National Institute for Health Research Cambridge Biomedical Research Centre, and Wellcome Strategic Award (100574/Z/12/Z). Medtronic is supplying discounted CGM devices, sensors, and details of communication protocol to facilitate real-time connectivity. Abbott Diabetes Care is providing Libre Pro sensors.

Relevant disclosures

RH reports having received speaker honoraria from Eli Lilly and Novo Nordisk, serving on advisory panel for Eli Lilly and Novo Nordisk, receiving licence fees from BBraun and Medtronic. RH and MEW report patent patents and patent applications.

MT has received speaker honoraria from Medtronic and NovoNordisk.

RPW reports receiving speaker honoraria from Dexcom and serving on advisory panels for Eli Lilly and Novo Nordisk and research support from Bigfoot Biomedical, Dexcom, Lexicon, Mannkind and Novo Nordisk. BAB is on Advisory Boards for Novo-Nordisk and Convatec, has received research support from Medtronic Diabetes, Tandem Diabetes, Insulet, Convatec, and Dexcom. SAW has received speaker honoraria from Medtronic, Insulet, and Tandem, and has received consultant honoraria from Sanofi and Zealand Pharmaceuticals.

KKH has received research support from Dexcom, Inc for an investigator-initiated project; he has received consultant fees from Lilly Innovation Center, Bigfoot Biomedical, and Insulet, Inc. LADM reports grants from Medtronic.

GM, JMA, SH, CB, FC, LD, NT, CLA, DSF, CK, JS, SB, PC declare no competing financial interests exist.

Ethics and IRB approvals

East of England - Cambridge South Research Ethics Committee (UK), Jaeb Center for Health Research Institutional Review (IRB) Office (813-975-8690 or irb@jaeb.org) (USA).

Provenance and peer review

Not commissioned, internally peer reviewed.

Table 1. Inclusion and exclusion criteria

**Summary of inclusion criteria**
▪ Age ≥6 and <19 years<br>▪ Type 1 diabetes as defined by World Health Organization (34) for at least 1 year<br>▪ Use of an insulin pump for at least 3 months, with good knowledge of insulin self-adjustment by subject or caregiver as judged by the investigator<br>▪ Using U-100 rapid acting insulin analogues Aspart or Lispro only<br>▪ Willing to perform regular finger-prick blood glucose monitoring, with at least 4 blood glucose measurements per day<br>▪ Screening glycated haemoglobin ≥58 mmol/mol (7.5%) and ≤86mmol/mol (10%) based on analysis from local laboratory<br>▪ Literate in English<br>▪ Willing to wear continuous glucose sensor and closed-loop system at home<br>▪ Willing to follow study specific instructions<br>▪ Willing to upload pump and glucose sensor data at regular intervals<br>▪ Access to Wi-Fi<br>▪ Living with someone who is trained to administer glucagon and is able to seek emergency assistance
**Summary of exclusion criteria**
▪ Living alone<br>▪ Current use of any closed-loop system<br>▪ Any other physical or psychological disease likely to interfere with the normal conduct of the study and interpretation of the study results, as judged by the investigator<br>▪ Untreated coeliac disease, adrenal insufficiency, or untreated thyroid disease<br>▪ Current treatment with drugs known to interfere with glucose metabolism (e.g., systemic corticosteroids, non-selective beta-blockers and monoamine oxidase inhibitors, etc.)<br>▪ Known or suspected allergy to insulin<br>▪ Clinically significant nephropathy (estimated glomerular filtration rate <45ml/min) or on dialysis, neuropathy or active retinopathy (presence of maculopathy or proliferative changes), as judged by the investigator<br>▪ Recurrent incidents of severe hypoglycaemia (>1 episode) during the previous 6 months (adolescents: severe hypoglycaemia is defined as an event requiring assistance of another person to actively administer carbohydrates, glucagon, or take other corrective actions including episodes of hypoglycaemia severe enough to cause unconsciousness, seizures or attendance at hospital; children: severe hypoglycaemia is defined as an event associated with a seizure or loss of consciousness)<br>▪ Recurrent incidents of diabetic ketoacidosis (>1 episode) during the previous 6 months<br>▪ Unwilling to avoid regular use of acetaminophen<br>▪ Lack of reliable telephone facility for contact<br>▪ Total daily insulin dose ≥2 IU/kg/day and <15 IU/day<br>▪ Pregnancy, planned pregnancy, or breast feeding<br>▪ Severe visual or hearing impairment<br>▪ Seizure disorder<br>▪ Medically documented allergy towards the adhesive (glue) of plasters or unable to tolerate tape adhesive in the area of sensor placement<br>▪ Serious skin diseases (e.g., psoriasis vulgaris, bacterial skin diseases) located at places of the body likely to be used for localisation of the glucose sensor<br>▪ Abusing illicit drugs, prescription drugs or alcohol<br>▪ Use of pramlintide (Symlin), or other non-insulin glucose lowering agents including sulphonylureas, biguanides, DPP4-Inhibitors, GLP-1 analogues, SGLT-1/ 2 inhibitors at time of screening<br>▪ Shift work with working hours between 10pm and 8am<br>▪ Sickle cell disease, haemoglobinopathy, or has received red blood cell transfusion or erythropoietin within 3 months prior to time of screening<br>▪ Eating disorder such as anorexia or bulimia<br>▪ Employed by Medtronic Diabetes or with immediate family members employed by Medtronic Diabetes

For peer review only - http://bmjopen.bmj.com/site/about/guidelines.xhtml

Table 2. Schedule of study visits / phone contacts when the participant is randomised to day-and-night closed-loop combined with low glucose feature (intervention group)

	Visit/ Contact	Description	Start relative to previous / next Visit / Activity	Duration
**Run-in**	**Visit 1**	Recruitment visit: consent, HbA1c, screening bloods, urine pregnancy test, baseline surveys, blinded CGM training and insertion		1-4 hours
	**Visit 2**	Review of baseline bloods, pump settings and CGM data; adjustment of treatment	2 weeks after Visit 1 (+1 week); Run-in could be repeated	1-2 hours
**Training Period**	**Visit 3**	Randomisation, repeat HbA1c if Visit 3 and Visit 1 are >28 days apart, urine pregnancy test, study pump training and initiation, competency assessment	May coincide with Visit 2, within 8 weeks of Visit 1	3-4 hours
	**Visit 3a**	Real-time CGM training and initiation, competency assessment	Within 0 to 7 days of Visit 3 (Visit 3a may coincide with Visit 3; training visits can be repeated)	2-4 hours
**CL + LGS Intervention (6 months)**	**Visit 4***	CL initiation at clinic/home: data download, CL and low glucose feature training, competency assessment, blinded CGM	4 weeks after Randomisation (±1 week)	2-6 hours
	Contact 1	Review use of study devices; study update	Within 24 to 48 hours after Visit 4	<1 hour
	**Visit 5****	Review use of study devices; study update	1 week after Visit 4 (± 3 days)	<1 hour
	Contact 2	Review use of study devices; study update	2 weeks after Visit 4 (±3 days)	<1 hour
	Contact 3	Review use of study devices; study update	1 month after Visit 4 (±2 weeks)	<1 hour
	Contact 4	Review use of study devices; study update	2 months after Visit 4 (±2 weeks)	<1 hour
	**Visit 6**	3-month visit: HbA1c, urine pregnancy test, data download, blinded CGM, surveys	4 months after Randomisation (±2 weeks)	1-3 hours
	Contact 5	Review use of study devices; study update	5 months after Randomisation (±2 weeks)	<1 hour
	Contact 6	Review use of study devices; study update	6 months after Randomisation (±2 weeks)	<1 hour
	**Visit 7**	Blinded CGM	2-4 weeks before planned Visit 8	<0.5 hour
	**Visit 8**	End of closed-loop treatment arm (6 months of CL): HbA1c, data download, surveys and focus groups; resume usual pump therapy	7 months after Randomisation (±2 weeks)	1-3 hours

* In-person clinic visit mandatory in USA only.

For peer review only - http://bmjopen.bmj.com/site/about/guidelines.xhtml

** Could be done via phone/e-mail in UK. In-person visit mandatory in USA only.
HbA1c, glycated haemoglobin; CGM, continuous glucose monitoring; CL, closed-loop.

For peer review only - http://bmjopen.bmj.com/site/about/guidelines.xhtml

Table 3. Schedule of study visits / phone contacts when the participant is randomised to usual care (conventional or sensor-augmented insulin pump therapy) (control group)

	Visit/ Contact	Description	Start relative to previous / next Visit / Activity	Duration
**Run-in**	**Visit 1**	Recruitment visit: consent, HbA1c, screening bloods, urine pregnancy test, baseline surveys, blinded CGM training and insertion		1-4 hours
	**Visit 2**	Review of baseline bloods, pump settings and CGM data; adjustment of treatment	2 weeks after Visit 1 (+1 week); Run-in could be repeated	1-2 hours
**Training period**	**Visit 3**	Randomisation, repeat HbA1c if Visit 3 and Visit 1 are >28 days apart, urine pregnancy test, insulin pump refresher training, competency assessment	May coincide with Visit 2, within 8 weeks of Visit 1	3-4 hours
**Usual insulin pump therapy Intervention (6 months)**	**Visit 4***	Initiation of standard therapy arm at clinic/home, glucometer download, recording of current insulin requirements, blinded CGM	4 weeks after Randomisation (±1 week)	2-6 hours
	Contact 1	Study update	Within 24 to 48 hours after Visit 4	<1 hour
	**Visit 5****	Study update	1 week after Visit 4 (±3 days)	<1 hour
	Contact 2	Study update	2 weeks after Visit 4 (±3 days)	<1 hour
	Contact 3	Study update	1 month after Visit 4 (±2 weeks)	<1 hour
	Contact 4	Study update	2 months after Visit 4 (±2 weeks)	<1 hour
	**Visit 6**	3-month visit: HbA1c, urine pregnancy test, glucometer download, recording of current insulin requirements, surveys, blinded CGM	4 months after Randomisation (±2 weeks)	1-3 hours
	Contact 5	Study update	5 months after Randomisation (±2 weeks)	<1 hour
	Contact 6	Study update	6 months after Randomisation (±2 weeks)	<1 hour
	**Visit 7**	Blinded CGM	2-4 weeks before planned Visit 8	<0.5 hour
	**Visit 8**	End of standard pump therapy treatment arm (6 months): HbA1c, glucometer download, recording of current insulin requirements, surveys and focus groups, resume usual care	7 months after Randomisation (±2 weeks)	1-3 hours

* In-person clinic visit mandatory in USA only.
** Could be done via phone/e-mail.
HbA1c, glycated haemoglobin; CGM, continuous glucose monitoring.

For peer review only - http://bmjopen.bmj.com/site/about/guidelines.xhtml

Figure Legends

Figure 1 Study flow chart. HbA1c, glycated haemoglobin; CGM, continuous glucose monitoring.

Figure 2 FlorenceM closed-loop system prototype.

The system consists of a continuous glucose monitoring transmitter with Guardian 3 sensor (Medtronic), an insulin pump (modified 640G pump, Medtronic), and an Android smartphone running the control algorithm (Cambridge).

For peer review only - http://bmjopen.bmj.com/site/about/guidelines.xhtml

References

1. International Diabetes Federation. IDF Diabetes Atlas 2017. 8th Edition 2017 http://www.diabetesatlas.org (last accessed 26th July 2018).
2. Wood JR, Miller KM, Maahs DM, Beck RW, DiMeglio LA, Libman IM, et al. Most youth with type 1 diabetes in the T1D Exchange Clinic Registry do not meet American Diabetes Association or International Society for Pediatric and Adolescent Diabetes clinical guidelines. Diabetes Care. 2013;36(7):2035-7.
3. Diabetes Research in Children Network Study G, Buckingham B, Beck RW, Tamborlane WV, Xing D, Kollman C, et al. Continuous glucose monitoring in children with type 1 diabetes. J Pediatr. 2007;151(4):388-93, 93 e1-2.
4. Bode BW, Schwartz S, Stubbs HA, Block JE. Glycemic characteristics in continuously monitored patients with type 1 and type 2 diabetes: normative values. Diabetes Care. 2005;28(10):2361-6.
5. Cryer PE. Diverse causes of hypoglycemia-associated autonomic failure in diabetes. N Engl J Med. 2004;350(22):2272-9.
6. Cryer PE. Mechanisms of hypoglycemia-associated autonomic failure and its component syndromes in diabetes. Diabetes. 2005;54(12):3592-601.
7. Wild D, von Maltzahn R, Brohan E, Christensen T, Clauson P, Gonder-Frederick L. A critical review of the literature on fear of hypoglycemia in diabetes: Implications for diabetes management and patient education. Patient Educ Couns. 2007;68(1):10-5.
8. Kropff J, DeVries JH. Continuous Glucose Monitoring, Future Products, and Update on Worldwide Artificial Pancreas Projects. Diabetes Tech Ther. 2016;18 Suppl 2:S253-63.
9. Juvenile Diabetes Research Foundation Continuous Glucose Monitoring Study G, Tamborlane WV, Beck RW, Bode BW, Buckingham B, Chase HP, et al. Continuous glucose monitoring and intensive treatment of type 1 diabetes. N Engl J Med. 2008;359(14):1464-76.
10. Kordonouri O, Pankowska E, Rami B, Kapellen T, Coutant R, Hartmann R, et al. Sensor-augmented pump therapy from the diagnosis of childhood type 1 diabetes: results of the Paediatric Onset Study (ONSET) after 12 months of treatment. Diabetologia. 2010;53(12):2487-95.
11. Pickup JC, Freeman SC, Sutton AJ. Glycaemic control in type 1 diabetes during real time continuous glucose monitoring compared with self monitoring of blood glucose: meta-analysis of randomised controlled trials using individual patient data. Bmj. 2011;343:d3805.
12. Bergenstal RM, Tamborlane WV, Ahmann A, Buse JB, Dailey G, Davis SN, et al. Effectiveness of sensor-augmented insulin-pump therapy in type 1 diabetes. N Engl J Med. 2010;363(4):311-20.
13. Bergenstal RM, Klonoff DC, Garg SK, Bode BW, Meredith M, Slover RH, et al. Threshold-based insulin-pump interruption for reduction of hypoglycemia. N Engl J Med. 2013;369(3):224-32.
14. Thabit H, Hovorka R. Coming of age: the artificial pancreas for type 1 diabetes. Diabetologia. 2016;59(9):1795-805.
15. Hovorka R. Closed-loop insulin delivery: from bench to clinical practice. Nat Rev Endocrinol. 2011;7(7):385-95.
16. FDA News Release. FDA approves first automated insulin delivery device for type 1 diabetes. http://www.fda.gov/NewsEvents/Newsroom/PressAnnouncements/ucm522974.htm (last accessed 26 July 2018) [
17. Kropff J, Del Favero S, Place J, Toffanin C, Visentin R, Monaro M, et al. 2 month evening and night closed-loop glucose control in patients with type 1 diabetes under free-living conditions: a randomised crossover trial. Lancet Diabetes Endocrinol. 2015;3(12):939-47.
18. Hovorka R, Allen JM, Elleri D, Chassin LJ, Harris J, Xing D, et al. Manual closed-loop insulin delivery in children and adolescents with type 1 diabetes: a phase 2 randomised crossover trial. Lancet. 2010;375(9716):743-51.
19. Elleri D, Allen JM, Kumareswaran K, Leelarathna L, Nodale M, Caldwell K, et al. Closed-loop basal insulin delivery over 36 hours in adolescents with type 1 diabetes: randomized clinical trial. Diabetes Care. 2013;36(4):838-44.

For peer review only - http://bmjopen.bmj.com/site/about/guidelines.xhtml

20. Nimri R, Danne T, Kordonouri O, Atlas E, Bratina N, Biester T, et al. The "Glucositter" overnight automated closed loop system for type 1 diabetes: a randomized crossover trial. Pediatr Diabetes. 2013;14(3):159-67.
21. Hovorka R, Elleri D, Thabit H, Allen JM, Leelarathna L, El-Khairi R, et al. Overnight Closed Loop Insulin Delivery in Young People with Type 1 Diabetes: A Free-Living Randomised Clinical Trial. Diabetes Care. 2014;37(5):1204-11.
22. Thabit H, Tauschmann M, Allen JM, Leelarathna L, Hartnell S, Wilinska ME, et al. Home Use of an Artificial Beta Cell in Type 1 Diabetes. N Engl J Med. 2015;373(22):2129-40.
23. Nimri R, Muller I, Atlas E, Miller S, Kordonouri O, Bratina N, et al. Night glucose control with MD-Logic artificial pancreas in home setting: a single blind, randomized crossover trial-interim analysis. Pediatr Diabetes. 2014;15(2):91-9.
24. Tauschmann M, Allen JM, Wilinska ME, Thabit H, Stewart Z, Cheng P, et al. Day-and-Night Hybrid Closed-Loop Insulin Delivery in Adolescents With Type 1 Diabetes: A Free-Living, Randomized Clinical Trial. Diabetes Care. 2016;Jan 6 [Epub ahead of print].
25. Leelarathna L, Dellweg S, Mader JK, Allen JM, Benesch C, Doll W, et al. Day and night home closed-loop insulin delivery in adults with type 1 diabetes: three-center randomized crossover study. Diabetes Care. 2014;37(7):1931-7.
26. Thabit H, Lubina-Solomon A, Stadler M, Leelarathna L, Walkinshaw E, Pernet A, et al. Home use of closed-loop insulin delivery for overnight glucose control in adults with type 1 diabetes: a 4-week, multicentre, randomised crossover study. Lancet Diabetes Endocrinol. 2014;2(9):701-9.
27. Barnard KD, Wysocki T, Allen JM, Elleri D, Thabit H, Leelarathna L, et al. Closing the loop overnight at home setting: psychosocial impact for adolescents with type 1 diabetes and their parents. BMJ Open Diabetes Res Care. 2014;2(1):e000025.
28. Bekiari E, Kitsios K, Thabit H, Tauschmann M, Athanasiadou E, Karagiannis T, et al. Artificial pancreas treatment for outpatients with type 1 diabetes: systematic review and meta-analysis. Bmj. 2018;361:k1310.
29. Wilinska ME, Chassin LJ, Acerini CL, Allen JM, Dunger DB, Hovorka R. Simulation environment to evaluate closed-loop insulin delivery systems in type 1 diabetes. J Diabetes Sci Technol. 2010;4(1):132-44.
30. Boughton CK, Hovorka R. Is an artificial pancreas (closed-loop system) for Type 1 diabetes effective? Diabet Med. 2019;36(3):279-86.
31. Stevens K. Developing a descriptive system for a new preference-based measure of health-related quality of life for children. Qual Life Res. 2009;18(8):1105-13.
32. Wille N, Badia X, Bonsel G, Burstrom K, Cavrini G, Devlin N, et al. Development of the EQ-5D-Y: a child-friendly version of the EQ-5D. Qual Life Res. 2010;19(6):875-86.
33. Effectiveness of continuous glucose monitoring in a clinical care environment: evidence from the Juvenile Diabetes Research Foundation continuous glucose monitoring (JDRF-CGM) trial. Diabetes Care. 2010;33(1):17-22.
34. World Health Organization. Definition, Diagnosis and Classification of Diabetes Mellitus and its Complications. Part 1: Diagnosis and Classification of Diabetes Mellitus. WHO/NCD/NCS/99.2. Geneva, (1999).

For peer review only - http://bmjopen.bmj.com/site/about/guidelines.xhtml

Figure 1. Study flow chart

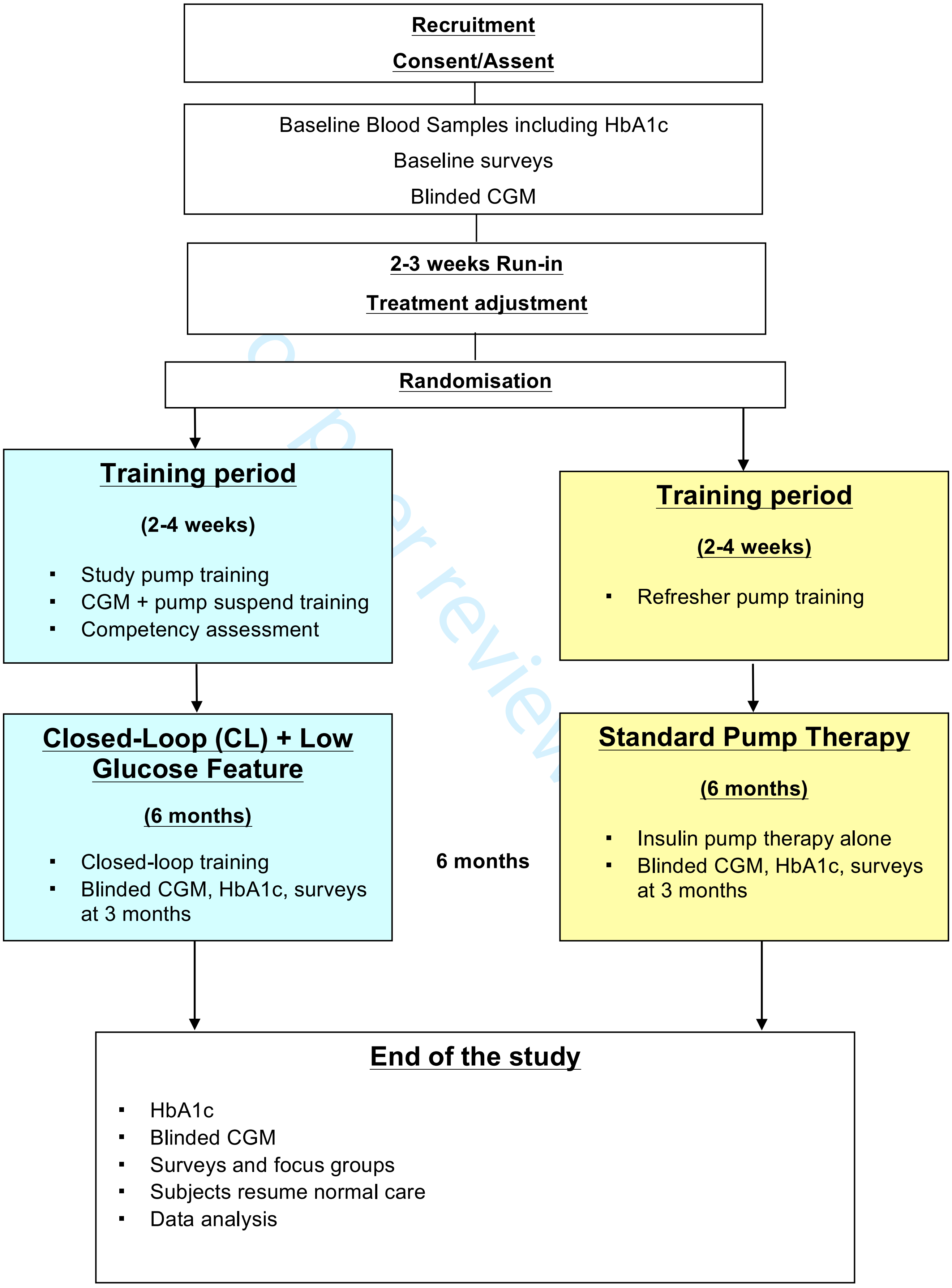

For peer review only - http://bmjopen.bmj.com/site/about/guidelines.xhtml

Figure 2. FlorenceM closed-loop system prototype

The system consists of a continuous glucose monitoring transmitter with Guardian 3 sensor (Medtronic), an insulin pump (modified 640G pump, Medtronic), and an Android smartphone running the control algorithm (Cambridge).

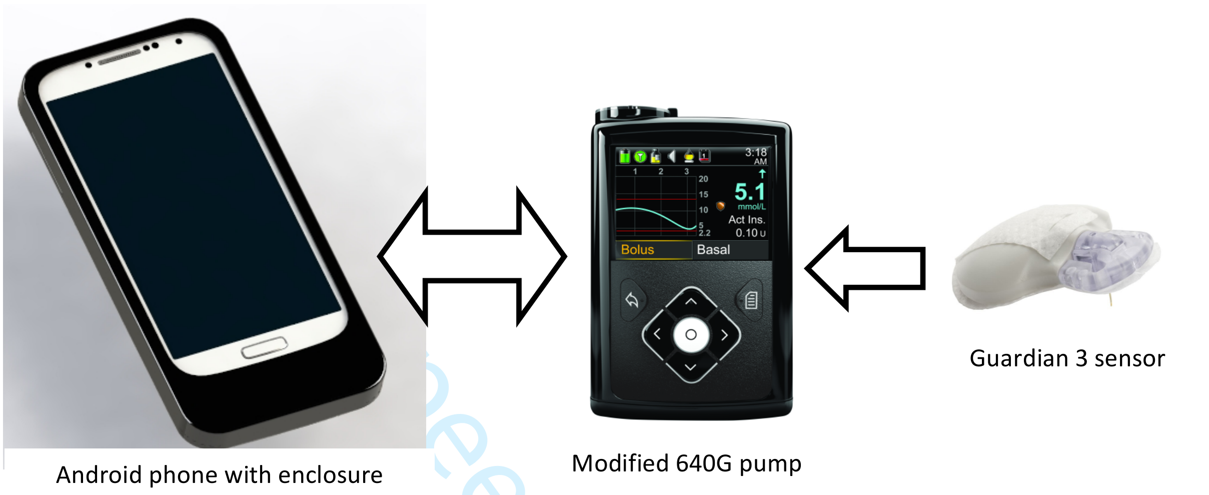

Reporting checklist for protocol of a clinical trial.

Based on the SPIRIT guidelines.

Instructions to authors

Complete this checklist by entering the page numbers from your manuscript where readers will find each of the items listed below.

Your article may not currently address all the items on the checklist. Please modify your text to include the missing information. If you are certain that an item does not apply, please write "n/a" and provide a short explanation.

Upload your completed checklist as an extra file when you submit to a journal.

In your methods section, say that you used the SPIRIT reporting guidelines, and cite them as:

Chan A-W, Tetzlaff JM, Altman DG, Laupacis A, Gøtzsche PC, Krleža-Jerić K, Hróbjartsson A, Mann H, Dickersin K, Berlin J, Doré C, Parulekar W, Summerskill W, Groves T, Schulz K, Sox H, Rockhold FW, Rennie D, Moher D. SPIRIT 2013 Statement: Defining standard protocol items for clinical trials. Ann Intern Med. 2013;158(3):200-207

		Reporting Item	Page Number
Title	#1	Descriptive title identifying the study design, population, interventions, and, if applicable, trial acronym	1
Trial registration	#2a	Trial identifier and registry name. If not yet registered, name of intended registry	2

For peer review only

			NCT02925299 (ClinicalTrials.gov) DAN05
Trial registration: data set	#2b	All items from the World Health Organization Trial Registration Data Set	04/10/2016
Protocol version	#3	Date and version identifier	16.04.2018 (6.0)
Funding	#4	Sources and types of financial, material, and other support	14
Roles and responsibilities: contributorship	#5a	Names, affiliations, and roles of protocol contributors	14
Roles and responsibilities: sponsor contact information	#5b	Name and contact information for the trial sponsor	NCT02925299 (ClinicalTrials.gov)
Roles and responsibilities: sponsor and funder	#5c	Role of study sponsor and funders, if any, in study design; collection, management, analysis, and interpretation of data; writing of the report; and the decision to submit the report for publication, including whether they will have ultimate authority over any of these activities	NCT02925299 (ClinicalTrials.gov)

For peer review only

Roles and responsibilities: committees	#5d	Composition, roles, and responsibilities of the coordinating centre, steering committee, endpoint adjudication committee, data management team, and other individuals or groups overseeing the trial, if applicable (see Item 21a for data monitoring committee)	See supplementary file
Background and rationale	#6a	Description of research question and justification for undertaking the trial, including summary of relevant studies (published and unpublished) examining benefits and harms for each intervention	4
Background and rationale: choice of comparators	#6b	Explanation for choice of comparators	4
Objectives	#7	Specific objectives or hypotheses	2, 5
Trial design	#8	Description of trial design including type of trial (eg, parallel group, crossover, factorial, single group), allocation ratio, and framework (eg, superiority, equivalence, non-inferiority, exploratory)	5
Study setting	#9	Description of study settings (eg, community clinic, academic hospital) and list of countries where data will be collected. Reference to where list of study sites can be obtained	5

For peer review only

Eligibility criteria	#10	Inclusion and exclusion criteria for participants. If applicable, eligibility criteria for study centres and individuals who will perform the interventions (eg, surgeons, psychotherapists)	15
Interventions: description	#11a	Interventions for each group with sufficient detail to allow replication, including how and when they will be administered	5, 16, 17
Interventions: modifications	#11b	Criteria for discontinuing or modifying allocated interventions for a given trial participant (eg, drug dose change in response to harms, participant request, or improving / worsening disease)	See protocol
Interventions: adherence	#11c	Strategies to improve adherence to intervention protocols, and any procedures for monitoring adherence (eg, drug tablet return; laboratory tests)	N/A
Interventions: concomitant care	#11d	Relevant concomitant care and interventions that are permitted or prohibited during the trial	7
Outcomes	#12	Primary, secondary, and other outcomes, including the specific measurement variable (eg, systolic blood pressure), analysis metric (eg, change from baseline, final value, time to event), method of aggregation (eg, median, proportion), and time point for each outcome.	9, 10

For peer review only

		Explanation of the clinical relevance of chosen efficacy and harm outcomes is strongly recommended	
Participant timeline	#13	Time schedule of enrolment, interventions (including any run-ins and washouts), assessments, and visits for participants. A schematic diagram is highly recommended (see Figure)	See Figure 1
Sample size	#14	Estimated number of participants needed to achieve study objectives and how it was determined, including clinical and statistical assumptions supporting any sample size calculations	12
Recruitment	#15	Strategies for achieving adequate participant enrolment to reach target sample size	5
Allocation: sequence generation	#16a	Method of generating the allocation sequence (eg, computer-generated random numbers), and list of any factors for stratification. To reduce predictability of a random sequence, details of any planned restriction (eg, blocking) should be provided in a separate document that is unavailable to those who enrol participants or assign interventions	6

For peer review only

Allocation concealment mechanism	#16b	Mechanism of implementing the allocation sequence (eg, central telephone; sequentially numbered, opaque, sealed envelopes), describing any steps to conceal the sequence until interventions are assigned	6
Allocation: implementation	#16c	Who will generate the allocation sequence, who will enrol participants, and who will assign participants to interventions	
Blinding (masking)	#17a	Who will be blinded after assignment to interventions (eg, trial participants, care providers, outcome assessors, data analysts), and how	N/A
Blinding (masking): emergency unblinding	#17b	If blinded, circumstances under which unblinding is permissible, and procedure for revealing a participant's allocated intervention during the trial	N/A
Data collection plan	#18a	Plans for assessment and collection of outcome, baseline, and other trial data, including any related processes to promote data quality (eg, duplicate measurements, training of assessors) and a description of study instruments (eg, questionnaires, laboratory tests) along with their reliability and validity, if known. Reference to where data	See protocol

For peer review only

		collection forms can be found, if not in the protocol	
Data collection plan: retention	#18b	Plans to promote participant retention and complete follow-up, including list of any outcome data to be collected for participants who discontinue or deviate from intervention protocols	See protocol
Data management	#19	Plans for data entry, coding, security, and storage, including any related processes to promote data quality (eg, double data entry; range checks for data values). Reference to where details of data management procedures can be found, if not in the protocol	12
Statistics: outcomes	#20a	Statistical methods for analysing primary and secondary outcomes. Reference to where other details of the statistical analysis plan can be found, if not in the protocol	9
Statistics: additional analyses	#20b	Methods for any additional analyses (eg, subgroup and adjusted analyses)	11
Statistics: analysis population and missing data	#20c	Definition of analysis population relating to protocol non-adherence (eg, as randomised analysis), and any statistical methods to handle missing data (eg, multiple imputation)	11

For peer review only

Data monitoring: formal committee	#21a	Composition of data monitoring committee (DMC); summary of its role and reporting structure; statement of whether it is independent from the sponsor and competing interests; and reference to where further details about its charter can be found, if not in the protocol. Alternatively, an explanation of why a DMC is not needed	12
Data monitoring: interim analysis	#21b	Description of any interim analyses and stopping guidelines, including who will have access to these interim results and make the final decision to terminate the trial	11
Harms	#22	Plans for collecting, assessing, reporting, and managing solicited and spontaneously reported adverse events and other unintended effects of trial interventions or trial conduct	12-13
Auditing	#23	Frequency and procedures for auditing trial conduct, if any, and whether the process will be independent from investigators and the sponsor	12
Research ethics approval	#24	Plans for seeking research ethics committee / institutional review board (REC / IRB) approval	13
Protocol amendments	#25	Plans for communicating important protocol modifications (eg, changes to eligibility criteria,	13

For peer review only

		outcomes, analyses) to relevant parties (eg, investigators, REC / IRBs, trial participants, trial registries, journals, regulators)	
Consent or assent	#26a	Who will obtain informed consent or assent from potential trial participants or authorised surrogates, and how (see Item 32)	5
Consent or assent: ancillary studies	#26b	Additional consent provisions for collection and use of participant data and biological specimens in ancillary studies, if applicable	N/A
Confidentiality	#27	How personal information about potential and enrolled participants will be collected, shared, and maintained in order to protect confidentiality before, during, and after the trial	12
Declaration of interests	#28	Financial and other competing interests for principal investigators for the overall trial and each study site	14
Data access	#29	Statement of who will have access to the final trial dataset, and disclosure of contractual agreements that limit such access for investigators	12
Ancillary and post trial care	#30	Provisions, if any, for ancillary and post-trial care, and for compensation to those who suffer harm from trial participation	N/A

For peer review only

Dissemination policy: trial results	#31a	Plans for investigators and sponsor to communicate trial results to participants, healthcare professionals, the public, and other relevant groups (eg, via publication, reporting in results databases, or other data sharing arrangements), including any publication restrictions	9, 13
Dissemination policy: authorship	#31b	Authorship eligibility guidelines and any intended use of professional writers	See supplementary file
Dissemination policy: reproducible research	#31c	Plans, if any, for granting public access to the full protocol, participant-level dataset, and statistical code	
Informed consent materials	#32	Model consent form and other related documentation given to participants and authorised surrogates	Approved consents for UK and USA available
Biological specimens	#33	Plans for collection, laboratory evaluation, and storage of biological specimens for genetic or molecular analysis in the current trial and for future use in ancillary studies, if applicable	8

The SPIRIT checklist is distributed under the terms of the Creative Commons Attribution License CC-BY-ND 3.0. This checklist can be completed online using https://www.goodreports.org/, a tool made by the EQUATOR Network in collaboration with Penelope.ai

For peer review only
